# EYA4 promotes breast cancer progression and metastasis through its role in replication stress avoidance

**DOI:** 10.1186/s12943-023-01861-4

**Published:** 2023-09-30

**Authors:** Bárbara de la Peña Avalos, Romain Tropée, Pascal H. G. Duijf, Eloïse Dray

**Affiliations:** 1 School of Biomedical Sciences, Faculty of Health, Queensland University of Technology, Brisbane, QLD Australia; 2 Present Address: Department of Biochemistry and Structural Biology, University of Texas Health Science Center at San Antonio, San Antonio, TX USA; 3 Present Address: Greehey Children’s Cancer Research Institute, University of Texas Health Science Center at San Antonio, San Antonio, TX USA; 4 Mays Cancer Center at UT Health San Antonio MD Anderson, San Antonio, TX USA; 5 Present Address: Southern RNA, Springfield Central, QLD 4300 Australia; 6 Centre for Cancer Biology, Clinical and Health Sciences, & SA Pathology, University of South Australia, Adelaide, SA Australia; 7 Institute of Clinical Medicine, Faculty of Medicine, University of Oslo, Oslo, Norway; 8 Department of Medical Genetics, Oslo University Hospital, Oslo, Norway

**Keywords:** EYA4, Protein phosphatase, Cell cycle, DNA replication, Breast cancer

## Abstract

The Eyes Absent (EYA) family of proteins is an atypical group of four dual-functioning protein phosphatases (PP), which have been linked to many vital cellular processes and organogenesis pathways. The four family members of this PP family possess transcriptional activation and phosphatase functions, with serine/threonine and tyrosine phosphatase domains. EYA4 has been associated with several human cancers, with tumor-suppressing and tumor-promoting roles. However, EYA4 is the least well-characterized member of this unique family of PP, with its biological functions and molecular mechanisms in cancer progression, particularly in breast cancer, still largely unknown. In the present study, we found that the over-expression of EYA4 in breast tissue leads to an aggressive and invasive breast cancer phenotype, while the inhibition of EYA4 reduced tumorigenic properties of breast cancer cells in vitro and in vivo. Cellular changes downstream of EYA4, including cell proliferation and migration, may explain the increased metastatic power of breast cancer cells over-expressing EYA4. Mechanistically, EYA4 prevents genome instability by inhibiting the accumulation of replication-associated DNA damage. Its depletion results in polyploidy as a consequence of endoreplication, a phenomenon that can occur in response to stress. The absence of EYA4 leads to spontaneous replication stress characterized by the activation of the ATR pathway, sensitivity to hydroxyurea, and accumulation of endogenous DNA damage as indicated by increased γH2AX levels. In addition, we show that EYA4, specifically its serine/threonine phosphatase domain, plays an important and so far, unexpected role in replication fork progression. This phosphatase activity is essential for breast cancer progression and metastasis. Taken together, our data indicate that EYA4 is a novel potential breast cancer oncogene that supports primary tumor growth and metastasis. Developing therapeutics aimed at the serine/threonine phosphatase activity of EYA4 represents a robust strategy for killing breast cancer cells, to limit metastasis and overcome chemotherapy resistance caused by endoreplication and genomic rearrangements.

**Supplementary Information:**

The online version contains supplementary material available at 10.1186/s12943-023-01861-4.

## Background

The Eyes Absent family (EYA1-4) is a unique group of dual-functioning protein phosphatases, which have been shown to promote cell proliferation, invasion, migration, and survival in a variety of cancers [1–3]. Members of the EYA family possess N-terminal transcriptional co-activation and threonine phosphatase activity, and C-terminal tyrosine phosphatase activity [4–6]. The highly conserved C-terminal domain, also known as the EYA domain (ED), contains a haloacid dehalogenase (HAD) signature sequence, making them the only known HAD-family tyrosine phosphatases (Fig. S1A) [4]. As the founding members of a new class of non-thiol-based protein tyrosine phosphatases, EYAs have a unique active site, using aspartic acid rather than cysteine as the nucleophile, making these atypical phosphatases attractive targets for specific inhibition with small molecules. However, the biological functions and cellular targets of these dual-phosphatases remain largely unknown, particularly for EYA4.

Defects in EYA4 have been linked to different developmental disorders including hearing loss [7] and cardiomyopathy [8]. EYA4 has also been associated with cancer in various organs. In malignant peripheral nerve sheath tumors (MPNST) EYA4 is over-expressed [9], whilst it is down-regulated in esophageal adenocarcinoma [10, 11], hepatocellular carcinoma [12], lung cancer [13] and colorectal cancer [14], where the *EYA4* gene promoter has been found to be hypermethylated. Consistent with this, our group and collaborators identified *EYA4* as a potential novel breast cancer gene [15]. Specifically, our observation that EYA4 is hypermethylated in the first intron–exon junction particularly in triple-negative breast cancer patients when compared to matched normal samples, led us to pursue its role in carcinogenesis and its cellular functions. To do this, we inactivated or over-expressed EYA4 in a variety of cell lines and investigated the resulting phenotypes, including cell cycle progression and DNA replication efficiency.

Here, we show that EYA4 is over-expressed in breast cancer and that this increases proliferation and migration in breast cancer cells, features that are linked with aggressive breast cancer in vivo. The function of EYA4 in promoting breast cancer growth and metastasis is also supported by in vivo xenograft studies showing that silencing of EYA4 expression in MDA-MB-231 cells leads to reduced cancer burden and distant metastasis. Interestingly, we found that the serine/threonine phosphatase activity of EYA4, but not its tyrosine phosphatase, is essential for breast cancer progression and metastasis.

In cells, we uncovered that EYA4 depletion promotes endoreplication and consequently polyploidy, a phenomenon that can occur in response to stress [16, 17] and can cause drug resistance [18]. The absence of EYA4 leads to spontaneous replication stress characterized by activation of key cell cycle checkpoints (pChk1 and pChk2), sensitivity to hydroxyurea, and accumulation of endogenous DNA damage, as indicated by increased γH2AX levels. It is important to mention that EYA proteins, particularly EYA1 and 3, have been previously linked to DNA damage, through the dephosphorylation of H2AX on tyrosine 142 in response to DNA damage, which facilitates the phosphorylation on serine 139 (known as γH2AX), promoting efficient DNA repair rather than apoptosis [19].

Upon induction of replication stress by hydroxyurea in EYA4-depleted cells, enhanced levels of unresolved DNA breaks are observed, suggesting that EYA4 plays a crucial role in the repair of replication-associated DNA damage.

Taken together, our data indicate that EYA4 is a potential novel oncogene in breast cancer and could play a role in cell cycle maintenance. This makes EYA4 an attractive druggable target in cancer treatments, especially in triple-negative breast cancer, to limit metastasis and overcome chemotherapy resistance.

## Materials and methods

### Plasmids

MISSION TRC2 pLKO.5-Puro empty vector (EV) or *EYA4* shRNA constructs (shRNA1, TRCN0000244430; shRNA2, TRCN0000218273; shRNA3, TRCN0000244429) were obtained from Sigma-Aldrich. pcDNA3.1-nV5 *EYA4* full length (FL) and pDEST26-His *EYA4* FL were cloned in our laboratory and sent for sequencing. pcDNA3.1-Myc-His *EYA4* mutant (S/T phosphatase deficient and Y phosphatase deficient) were obtained from General Biosystems.

### Cell culture and maintenance, transfections, and stable cell line establishment

HeLa, MDA-MB-231 and MCF-7 cells were sourced from ATCC. Cells were cultured in Dulbecco’s Modified Eagle’s Medium (DMEM; Gibco) supplemented with 10% fetal bovine serum (FBS) at 37°C in 5% CO_2_ incubators and passaged at 80% confluence or less. MCF-7 cells were supplemented with 10 μg/mL insulin and 1 mM sodium pyruvate. 1.2 × 10^6^ HEK 293 T cells were reverse-transfected using Lipofectamine 2000 reagent (Invitrogen) with pLKO.5 empty vector or *EYA4* shRNA constructs and Lenti-vpak plasmids from OriGene to create lentivirus particles. Viruses were harvested at 48 and 72 h post-transfection, filtered through a 0.45 μm filter, and used to infect HeLa or MDA-MB-231 cells with 4 μg/mL polybrene. Stable cell lines were selected using 1–2 μg/mL of puromycin. For complementation, stable HeLa or MDA-MB-231 cells expressing shRNA1 were transfected with pcDNA3.1 Myc/His containing a mutant version of EYA4 in the S/T domain (Y281F, Y284F, Y285F; referred to as S/T phosphatase deficient) or Y domain (D375N, D377N, T548A, E606Q, E607Q, E608Q; referred to as Y phosphatase deficient) and selected with 500 μg/mL geneticin. MCF-7 cells were transfected with pcDNA3.1-nV5 *EYA4* FL or pDEST26-His *EYA4* FL to create over-expressing (OE) clones (called OE-1 and OE-2, respectively). These clones were selected with 500 μg/mL geneticin. MDA-MB-231/Luc and MCF-7/Luc cells stably expressing firefly luciferase were established as described above. HeLa cells were transduced with FUCCI (red/green) plasmids [20] and FACS sorted to select homogenous positive cell populations. The origin of all cells was confirmed by short-tandem repeat (STR) profiling and tested regularly for *Mycoplasma* contamination.

### RNA extraction and quantitative reverse transcription PCR (qRT-PCR)

Total RNA was isolated from transfected or transduced cells by phenol–chloroform extraction (TRIzol; Invitrogen) followed by nucleic acid precipitation. The GoScript Reverse Transcription System (Promega) was used to generate first-strand cDNA. Quantitative PCR was performed using TaqMan probes spanning across exons for human *EYA4* (Invitrogen Hs01012406_mH) and human *18S* (Invitrogen Hs99999901_s1) to amplify 70 bp and 187 bp fragments, respectively. The relative expression of *EYA4* was determined using the 2^−ΔΔCt^ method with *18S* as an endogenous control for normalization.

### Immunoblotting

Immunoblotting analysis was conducted according to our standard procedures [21]. Cells were collected and lysed in RIPA buffer (10 mM Tris–HCl (pH 8.0), 1 mM EDTA, 0.5 mM EGTA, 1% Triton X-100, 0.1% sodium deoxycholate, 0.1% SDS, 140 mM NaCl) supplemented with cOmplete Mini EDTA-free protease inhibitor cocktail (Roche), 1 mM PMSF, 1 mM Na_3_VO_4_, 1 mM NaF, 1 mM benzamidine and 0.025 U/μL benzonase, followed by sonication for 2 min (40%) in an ultrasonic water bath (Sonics Vibra-Cell VCX400). Proteins were resolved in 4–20% Mini-Protean TGX gels (Bio-Rad) and transferred to Immobilon-P PVDF membranes (Merck). Membranes were then blocked with either 5% skim milk or bovine serum albumin (BSA) in TBS-T. Blots were incubated with primary antibody at either 4°C overnight or room temperature (RT) for 2 h, washed, then incubated with secondary HRP-conjugated antibodies for 1 h at RT. Bands were visualized using the Clarity Western ECL substrate (Bio-Rad). Primary antibodies: EYA4 (Abcam ab93865), cyclin E1 (HE12; Cell Signaling #4129), CDK2 (78B2; Cell Signaling #2546), p21^WAF1/CIP1^ (12D1; Cell Signaling #2947), p27^KIP1^ (D69C12; Cell Signaling # 3686), cyclin A (B-8; Santa Cruz sc-271682), pChk1 (S345) (133D3; Cell Signaling # 2348), pChk2 (T68) (Cell Signaling # 2661), γH2AX (S139) (Millipore 05–636), PCNA (PC10; Santa Cruz sc-56), GAPDH (14C10; Cell Signaling #2118) and β-Actin (C4; Santa Cruz sc-47778).

### Immunohistochemistry on human tissues

Three available anti-EYA4 antibodies were considered to evaluate the expression of EYA4 in normal breast tissue and breast carcinoma: HPA004805, HPA038771 and HPA038772 [22]. Since only HPA038771 was orthogonally or independently validated by the Human Protein Atlas (HPA) for the purpose of immunohistochemistry (IHC), as previously described [22], this antibody was selected. Immunohistochemistry (IHC) using DAB (3,3'-diaminobenzidine)-labeled HPA038771 was conducted as previously described (https://www.proteinatlas.org) [22]. HPA staining results of normal breast tissue (*n* = 3; 1 replicate each) and breast carcinoma (*n* = 12; 2 replicates each) were included (Fig. S1D) and HPA-interpreted results of these were analyzed using a two-sided Fisher's exact test (Table S1). In addition, IHC images were evaluated using a more accurate and more widely accepted IHC quick-score method [23]. Briefly, for each sample, this method renders an H-score, which is the sum of (1) the product of 1 and the percentage of cells stained weakly positive, (2) the product of 2 and the percentage of cells stained intermediately positive, and (3) the product of 3 and the percentage of cells stained strongly positive. For each breast carcinoma sample, one H-score was obtained by averaging the H-scores of each of the two replicates. Statistical differences in H-scores between normal breast and breast carcinoma tissues and between lobular and ductal breast carcinomas were determined using a two-sided Mann–Whitney *U*-test and a two-sided student *t*-test with Welch's correction, respectively, since data respectively did not and did pass Shapiro–Wilk normality tests (*p* < 0.05 and *p* > 0.05).

### Subcutaneous tumor xenografts in immunodeficient mice

For subcutaneous injections, MCF-7/Luciferase wild type (WT), and EYA4 over-expressing cells (1.0 × 10^6^) were resuspended in 100 μL of 0.9% (w/v) NaCl and injected in the left mammary fat pad (MFP) of 24 non-obese diabetic/severe combined immunodeficiency gamma (NSG, NOD scid gamma) female mice (6 weeks of age; 8 mice per cell line). A 17β-estradiol pellet (1.7 mg/pellet, 60-days release, Innovative Research of America) was implanted close to the neck using a precision trochar, 24 h prior to MFP injections. Weekly, all mice were weighed, tumor growth was measured by using a caliper and detected in vivo by bioluminescent imaging. For in vivo imaging, mice were first injected with D-luciferin (150 mg/kg, 10 min prior to imaging), anesthetized with 3% isoflurane and then imaged in an IVIS spectrum imaging system (Caliper, Newton, USA). Images were analyzed with Living Image software (Caliper, Newton, USA). Bioluminescent flux (photons/sec/sr/cm^2^) was determined for the tumors. Tumor volume was calculated according to the following formula: (length × width^2^)/2. MCF-7/Luciferase mice were sacrificed before tumors reached 1 cm^3^ (8 weeks post-injection). Harvested tumor tissues were placed in liquid nitrogen and then frozen at -80 °C or fixed in 10% buffered formalin, embedded in paraffin, sectioned, and stained. Antibodies used: anti-Estrogen Receptor (SP1; Roche 790–4324; CC1 64 min), anti-Ki-67 (30–9; Roche 790–4286; CC1 64 min), anti-γH2AX (pS319; Abcam ab2893; CC1 64 min; 1:600).

### Mouse tail-vein assay

MDA-MB-231/Luciferase WT, EYA4 shRNA1 and EYA4 shRNA2 cells (1.0 × 10^6^ cells/100 μL 0.9% (w/v) NaCl) were injected in the lateral tail-vein of 9 female NOD scid gamma mice (6 weeks of age; 3 mice per cell line). For complementation, MDA-MB-231/Luciferase WT, EYA4 shRNA1, and EYA4 shRNA1 complemented with pcDNA3.1-Myc-His *EYA4* mutant (S/T phosphatase deficient or Y phosphatase deficient) cells (1.0 × 10^6^ cells/100 μL 0.9% (w/v) NaCl) were injected in the lateral tail-vein of 22 female NSG mice (7 weeks of age; 7 WT mice and 5 mice per cell line). Mice were detected every week for metastatic foci by bioluminescent imaging as described above. MDA-MB-231/Luciferase mice were monitored and culled 4–5 weeks post-injection. Bioluminescent flux (photons/s/cm^2^/sr) was determined. Organs in which metastatic foci were observed were harvested and fixed in 4% PFA, followed by 70% EtOH, then embedded in paraffin, sectioned, and stained. Antibodies used as described above.

### Cell proliferation assay

Cells were seeded in a 96-well plate at 2.0 × 10^3^ cells/well. Phase contrast images of cells were acquired every 2 h using an IncuCyte Zoom (Essen BioScience) live imaging system. Proliferation was measured as a percentage of confluency.

### In vitro migration assay

Cells were cultured in a 96-well plate for 24 h to achieve 100% confluency. An IncuCyte Woundmaker was used to make a scratch in the cell monolayer. Cells were then incubated in serum-free media and automatically imaged every 2 h using an IncuCyte Zoom (Essen BioScience) live imaging system. The scratch gap width and confluence were measured at each time point and compared between groups.

### Apoptosis

HeLa cells were seeded in a 96-well plate (100 cells/well). After 24 h, annexin V (red) reagent was added according to manufacturer’s protocol (IncuCyte). Images (phase contrast/orange) were acquired every 2 h using an IncuCyte SX5 (Sartorius) live-cell imaging system. Apoptosis was measured as total integrated intensity (OCU × μm^2^/image).

### Double thymidine block and cell cycle progression (flow cytometry)

HeLa cells were synchronized in early S-phase by a double thymidine block. Briefly, cells were blocked with 2 mM thymidine for 18 h, released for 9 h, and blocked again with 2 mM thymidine for 17 h. After the second block, cells (asynchronized and synchronized) were released and collected according to time points, then fixed in ice-cold 70% ethanol at -20°C for at least 24 h. DNA was stained with 38 mM trisodium citrate, 100 μg/mL RNase A and 150 μg/mL propidium iodide (PI) for 1 h at RT. A DNA control PI (trout erythrocytes) was used as an internal control to normalize the cell cycle. Data were collected using a CytoFLEX Flow Cytometer (Beckman Coulter) and cell cycle profiles were analyzed with FlowJo to determine the percentage of cells in G1, S and G2/M. 10,000 events were collected, and aggregated cells were gated out.

### FUCCI

HeLa FUCCI cells stably transfected with empty vector or EYA4 shRNAs were seeded in a 96-well plate (100 cells/well). Phase contrast and green/orange images were acquired every 2 h to monitor cell cycle progression using an IncuCyte SX5 (Sartorius) live-cell imaging system. Images were analyzed using cell-by-cell analysis software and population subsets were classified based on green and red fluorescence. G1 phase (red), G1-S transition (green + red), S/G2/M phase (green) and M-G1 transition (non-fluorescent) [20].

### Indirect immunofluorescence

Indirect immunofluorescence was performed as described elsewhere [24]. Cells were grown on coverslips for 24 h and treated with 4 Gy γ-irradiation (Gammacell40 Exactor unit) or 4 mM hydroxyurea. Cell nuclei were pre-extracted with nuclear extraction buffer (NEB; 10 mM PIPES (pH 6.8), 100 mM NaCl, 300 mM sucrose, 3 mM MgCl_2_, 1 mM EGTA (pH 8.0), 0.5% Triton X-100) for 2 min at RT then fixed with 4% paraformaldehyde (PFA) for 10 min at 4°C. Nuclei were blocked in 5% BSA and 0.3% Triton X-100 in PBS, immunoblotted with a primary antibody (1:500 in dilution buffer; 1% BSA and 0.3% Triton X-100 in PBS), followed by secondary antibody (2 μg/mL in dilution buffer). DNA was counterstained with DAPI. Slides were viewed on an Olympus FV3000 confocal microscope. Primary antibodies: CENP-F (H-260; Santa Cruz sc-22791), γH2AX (S139) (Millipore 05–636). Secondary antibodies: α-Rabbit (Abcam ab150081, Alexa Fluor 488), α-Mouse (Abcam ab150103, Alexa Fluor 647). Nuclear foci quantification was performed using CellProfiler.

### MTT cell cytotoxicity assay

For genotoxic stress, cells were seeded into 96-well plates (200 cells/well). Twenty-four hours after seeding, increasing concentrations of ATR inhibitor (AZ20) or hydroxyurea were added to the culture (24 h pulse). Cell cytotoxicity was measured after 96 h following manufacturer’s protocol (Abcam ab211091). Briefly, 50 μL serum-free media (no phenol red) and 50 μL MTT reagent was added to each well and incubated at 37°C for 3 h. MTT media was replaced with 150 μL of MTT solvent and incubated with agitation for 15 min. Absorbance was measured at 590 nm. The cell viability was calculated using the following equation:

$$Cell\;viability \left(\mathrm{\%}\right)= \frac{{OD}_{treated}}{{OD}_{control}} \times 100$$

OD_treated_ and OD_control_ represented the absorbance of sampled and control, respectively.

### EdU incorporation

HeLa control and EYA4 knockdown cells (4.0 × 10^4^ cells/well) were seeded in 12-well plates with coverslips for 24 h. 5-ethynyl-2’-deoxyuridine (EdU) incorporation was performed according to manufacturer’s protocol (Base Click). Briefly, cells were treated with 4 mM hydroxyurea for 2 h, released for 10 min, then labeled with 10 μM of EdU for 30 min at 37˚C, then fixed with 4% PFA for 10 min at 4 °C, followed by permeabilization with 0.3% Triton X-100 in PBS for 20 min at RT. Reaction cocktail with 6-FAM azide was added to fixed cells and incubated for 30 min at RT. DNA was counterstained with DAPI. Slides were viewed on an Olympus FV3000 confocal microscope. EdU-stained cells were quantified using CellProfiler.

### DNA fiber assay

DNA combing was performed as previously described [25]. Briefly, exponentially growing HeLa cells (3.0 × 10^5^) were labeled with a 5-iodo-2’-deoxyuridine (IdU; 50 μM) pulse for 30 min. After labeling, cells were harvested, embedded in agarose and DNA was prepared then combed onto silanized coverslips using the FiberComb Molecular Combing System (Genomic Vision). Following combing, coverslips were baked for 2 h at 65 °C. Combed DNA fibers were denatured with 0.5 M NaOH + 1 M NaCl for 8 min at RT, neutralized with PBS (3 times, 3 min), then dehydrated in ethanol (70%-90%-100%, 3 min each), and air-dried. Combed DNA was blocked with BlockAid blocking solution (Invitrogen B10710) for 15 min at RT, followed by immunostaining with mouse α-BrdU (to detect IdU; BD Biosciences 347580) for 1 h at 37 °C, washed with PBS-T, and probed with secondary antibody (α-mouse Cy3, SIGMA C2181) for 45 min at 37 °C. Single-stranded DNA was counterstained with α-ssDNA mouse antibody (DSHB University of Iowa) for 2 h at 37 °C, followed by α-mouse BV480 (Jackson ImmunoResearch 115–685–166) for 45 min at 37 °C. Coverslips were washed in PBS, subjected to a graded ethanol series, air-dried, and then mounted with 25 μL of Vectashield mounting medium (Vector Laboratories). DNA fiber images were acquired on an Olympus FV3000 confocal microscope. Track lengths were measured with ImageJ. To calculate replication fork speed, the following equation was used to convert fork length from μm to kb/min: length μm × 2/labeling time in min = fork speed kb/min (conversion factor of 2 kb/μm specific for DNA combing method).

### Statistical analysis

The statistical analyses were conducted using GraphPad Prism 9 and a *p* < 0.05 was considered statistically significant. Student’s *t*-test was used to test for significant differences between groups, considering a normal distribution. Unpaired two-tailed tests were applied to all data if not specified. Samples sizes were chosen according to previously published methods where significant biological conclusions were reported.

## Results

### EYA4 is over-expressed in breast cancer

**Fig. 1:**
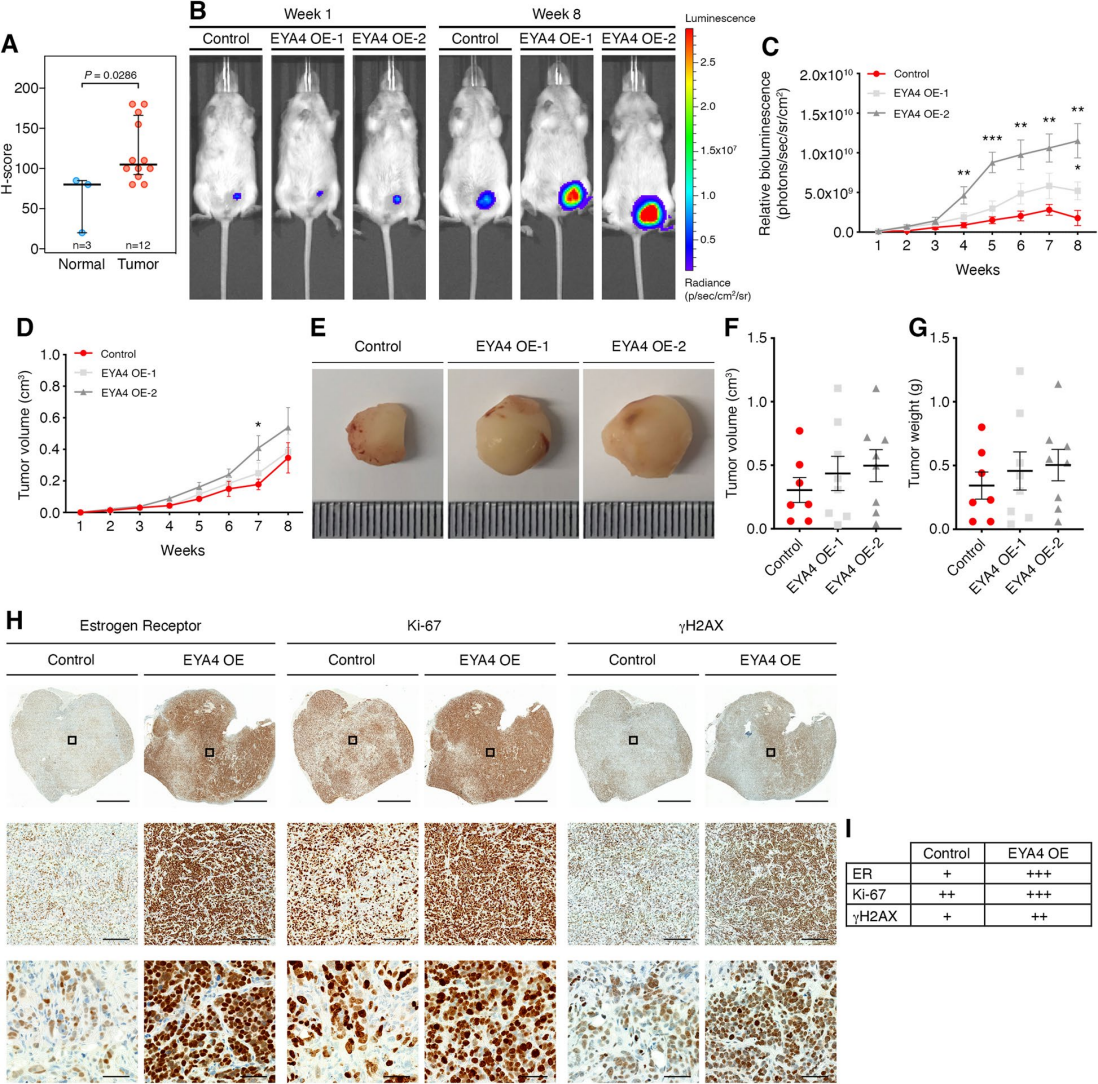
EYA4 is a potential novel breast cancer oncogene*.* **A** Immunohistochemistry-based H-score of normal breast tissues (*n* = 3) and breast carcinoma tissues (*n* = 12) using a validated anti-EYA4 antibody. Error bars show the medians with interquartile ranges. *P* value: Mann–Whitney *U*-test. **B**-**G** Measurement of tumor growth in xenograft mice bearing orthotopic MCF-7 control and EYA4 over-expressing tumors. **B**-**C** Tumor growth was monitored at the indicated time points by whole animal bioluminescence imaging (BLI). **B** Representative BLI signal of tumors is shown for weeks 1 and 8. **C** Quantification of the BLI signal of tumors is shown (mean ± SEM; *n* = 8). **D**-**G D** Tumor growth was also measured weekly using digital calipers (mean ± SEM; *n* = 8). **E** Representative images of surgically removed tumor mass are shown. Tumor (**F**) volume and (**G**) weight at week 8 are plotted (mean ± SEM; *n* = 8). **H**-**I** Immunohistochemistry staining of mouse MCF-7 tumors. **H **Representative images of surgically removed and stained tumors are shown. Low magnification (scale bar 2 mm), mid magnification (scale bar 200 μm), and high magnification views (scale bar 50 μm). Black boxes indicate the areas shown at higher magnification. **I** A score of negative (-), weak ( +), moderate (+ +) or strong (+ + +) was given to each stain. For all panels * *P* ≤ 0.05, ** *P* ≤ 0.01, *** *P* ≤ 0.001, except when indicated

We investigated whether EYA4 is expressed in breast cancer cell lines of various subtypes using real-time quantitative PCR and immunoblotting (Fig. S1B-C). The expression of EYA4 varied greatly across cell lines, however, the triple-negative breast cancer cell line MDA-MB-231 showed the highest endogenous expression of EYA4. We also assessed whether EYA4 is over-expressed in breast cancer tissues by immunohistochemistry using an orthogonally validated EYA4-specific antibody (see methods). We compared EYA4 expression in 3 normal breast epithelial tissues to 12 breast carcinoma tissues. Independent analysis [22] indicated that EYA4 is statistically significantly over-expressed in breast carcinoma (*P* = 0.0440, Fisher's exact test; Table S1). However, this method is rather crude. Therefore, we applied a more accurate and more widely accepted IHC quick-score method, yielding H-scores for each sample (see methods). This analysis also confirmed that EYA4 is over-expressed in breast carcinoma (*P* = 0.0286, Mann–Whitney *U*-test; Fig. 1A, Table S2, Fig. S1D). In addition, we observed that EYA4 expression is significantly higher in ductal than in lobular breast carcinomas (*P* = 0.0326, *t*-test; Fig. S1E), consistent with the observation that the 5-year mortality rate is significantly poorer for ductal than for lobular breast carcinoma patients [26]. Thus, EYA4 is over-expressed in breast carcinoma at the protein level and this is reflected in the MDA-MB-231 cell line.

### *EYA4 over-expression promotes breast cancer development *in vivo

In most mouse strains, knockout of *EYA4* is lethal shortly after birth and is toxic in several lung cancer cell lines [13, 27] and other cell lines that we tested. Using short-hairpin RNAs (shRNAs), EYA4 expression could be significantly decreased in MDA-MB-231 cells (Fig. S1F) or in HeLa cells (Fig. 3A). The most efficient hairpin, shRNA3, induces cell death in MDA-MB-231, indicating that EYA4 is essential in these cells. In parallel, we over-expressed EYA4 in the ER^+^/PR^+^ breast cancer cell line, MCF-7 (Fig. S1G), which expresses low or no detectable endogenous EYA4 ([28], Fig. S1B-C and Cancer Cell Line Encyclopedia, https://sites.broadinstitute.org/ccle). We assessed the effects of EYA4 deregulation on primary cancer growth and metastasis in vivo using luciferase-expressing cell lines. A human tumor xenograft model was established using NOD scid gamma mice. MCF-7/Luc wild type (WT) and EYA4 over-expressing (EYA4 OE-1 and EYA4 OE-2) cells were injected subcutaneously into the left mammary fat pad (MFP) of female mice supplemented with 17β-estradiol and monitored by caliper measurement and in vivo imaging for 8 weeks. Following an intraperitoneal injection with D-luciferin (150 mg/kg), the firefly luciferase enzyme catalyzes this substrate, which results in light photons that are captured by a charge-coupled device (CCD) camera mounted within an IVIS® Spectrum Imaging System [29]. As shown in Fig. 1B-C, the bioluminescence intensity (BLI) signal measurement confirmed tumor engraftment for all mice. Primary tumors show a significant increase in volume when EYA4 is over-expressed. BLI signal correlated with caliper measurements as observed in Fig. 1D, and with tumor volume and weight (Fig. 1F and G) once surgically removed postmortem (Fig. 1E). EYA4 over-expression leads to a more aggressive breast cancer, as observed by immunohistochemistry (IHC) staining (Fig. 1H). Our observations correspond with previous reports that in MPNST, EYA4 is dramatically upregulated in cells and primary tumors, and its depletion leads to reduced cell adhesion and migration in vitro and has an inhibitory effect in tumorigenesis in vivo [9].

Estrogen receptor alpha (ER-α) co-stain was used to validate human cells. Interestingly, cells expressing high levels of EYA4 also showed high expression of ER-α, the proliferation-related antigen Ki-67, and γH2AX, a marker of DNA damage (Fig. 1H-I). ER-α has a well-established role in supporting estrogen-dependent breast tumor growth through its association with aberrant proliferation (up-regulating Ki-67), which can result in the accumulation of random DNA mutations (marked by γH2AX), and when highly expressed it is associated with poor prognosis in breast cancer [30, 31], which can explain the aggressive breast cancer subtype observed when EYA4 is over-expressed.

**Fig. 2:**
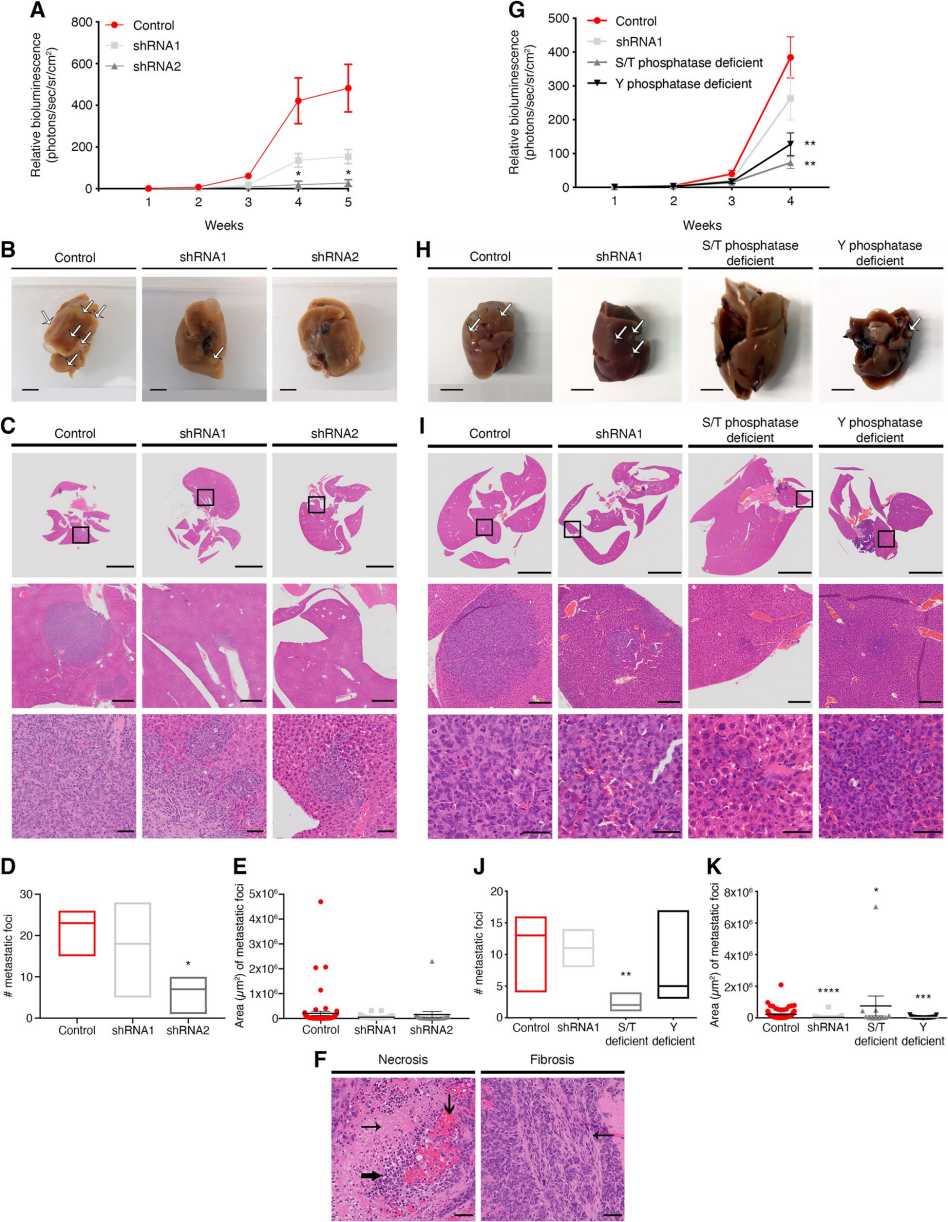
EYA4 promotes breast cancer development through its serine/threonine phosphatase domain***. ***Analysis of distant metastasis after mouse lateral tail-vein injections. **A** and **G **Metastasis was monitored at the indicated time points by whole animal bioluminescence imaging (BLI). Quantification of the BLI signal is plotted (mean ± SEM; *n* = 3 for A, and *n* = 5 for G). **B** and **H** Representative images of surgically removed livers (scale bar 5 mm) are shown. White arrows indicate liver metastatic foci. **C**-**E** and **I**-**K** Analysis of liver metastatic foci. **C** and **I** Hematoxylin and eosin staining of livers dissected 4–5 weeks post tail-vein injection. Low magnification (scale bar 5 mm), mid magnification (scale bar 500 μm), and high magnification views (scale bar 50 μm). Black boxes indicate the areas shown at higher magnification. Quantification (**D** and **J**; median; *n* = 3 for **D**, and *n* = 5 for **J**) and area (**E** and **K**; mean ± SEM) of liver metastatic foci are plotted. **F** Liver metastatic site with central necrosis (➞), accumulation of inflammatory cells (➨) and blood vessel congestion (➔), shown in left panel; and fibrosis (➞), shown in right panel. Scale bar 50 μm. For all panels * *P* ≤ 0.05, ** *P* ≤ 0.01, *** *P* ≤ 0.001, **** *P* ≤ 0.0001

Since breast cancer subtypes are associated with unique patterns of metastatic spread, we assessed metastatic capacity utilizing MDA-MB-231 stably expressing firefly luciferase. MDA-MB-231/Luciferase WT cells and cells in which EYA4 was stably knocked down (shRNA1 and shRNA2) were injected into the tail vein and monitored by in vivo imaging over 5 weeks. While WT and EYA4-depleted cells colonized the lungs as expected following systemic injection, we observed a decrease in BLI signal in mice injected with EYA4-depleted cells compared to the control (Fig. 2A). This was directly linked to a lesser number and a decrease in the area of metastatic foci observed in livers as revealed by histological analyses (Fig. 2B-E). Importantly, these IHC analyses also showed significant areas of central necrosis with inflammatory cells and blood vessel congestion (left panel) and scant fibrosis (right panel) was observed in the control group but not in the EYA4 knockdown mice (Fig. 2F). This particular observation could be due to the role that EYA4 plays in innate immune system regulation by enhancing the expression of IFN-β and CXCL10, in response to DNA stimulation [5]. In cancer cells, the cGAS-STING pathway is constitutively activated, inducing chronic IFN-β expression, triggered by the accumulation of DNA damage due to replication fork collapse or reactive oxygen species (ROS) that leads to the presence of DNA in the cytoplasm [32]. Altogether, our data suggest that EYA4 is a driver of breast cancer and that decreasing its expression reduces tumor and metastatic burdens.

### The S/T phosphatase domain of EYA4 contributes to breast cancer development

EYA4 possesses both serine/threonine (S/T) and tyrosine (Y) phosphatase activities (Fig. S1A) [6]. We designed EYA4 phosphatase mutants in the S/T domain (Y281F, Y284F, Y285F; henceforth, S/T phosphatase deficient) and in the Y domain (D375N, D377N, T548A, E606Q, E607Q, E608Q, referred to as Y phosphatase deficient), following published reports that identified the catalytic residues [5]. We confirmed that all constructs are expressed at a similar level in the cells tested, as evaluated by Western blotting (Fig. S3E and K). To investigate the relevance of these activities on tumor growth, MDA-MB-231/Luc EYA4-depleted cells complemented with either EYA4 S/T phosphatase deficient mutant or Y phosphatase deficient mutant were injected into the tail-vein and monitored by in vivo imaging for 4 weeks using the luciferase reporter. The phosphatase mutants (S/T phosphatase deficient and Y phosphatase deficient) caused even more significant outcomes that EYA4 depletion, especially the S/T phosphatase deficient mutant. Both EYA4 phosphatase mutants did not complement EYA4 depletion with shRNA1, as observed by both BLI signal (Fig. 2G) and by metastatic foci observed in livers (Fig. 2H-K). However, the serine/threonine phosphatase activity of EYA4 is the one that shows more significant outcomes, as observed not only by decreased tumor burden to lungs (Fig. S2C-E), but also by a lesser number of metastatic foci to the liver, with an average of 2 foci for S/T phosphatase deficient mutant, compared to 6 for EYA4 shRNA2 and 7 for Y phosphatase deficient mutant (Fig. 2J). In addition, as observed by IHC staining, when a metastatic site is observed (marked by H&E) in mouse injected with S/T phosphatase deficient mutant cells, there is no stain by Ki-67 or γH2AX (Fig. S2F). For γH2AX, only a background level (mouse cells stained), can be observed. Notably, all mice injected with S/T phosphatase deficient mutant cells showed liver enlargement and hyperplasia (Fig. 2H-I), which could be driven by an increased hepatocyte number, prompting further investigation. These data suggest that the serine/threonine phosphatase activity of EYA4 is essential for breast cancer progression and metastasis.

### EYA4 promotes cell proliferation and migration

**Fig. 3:**
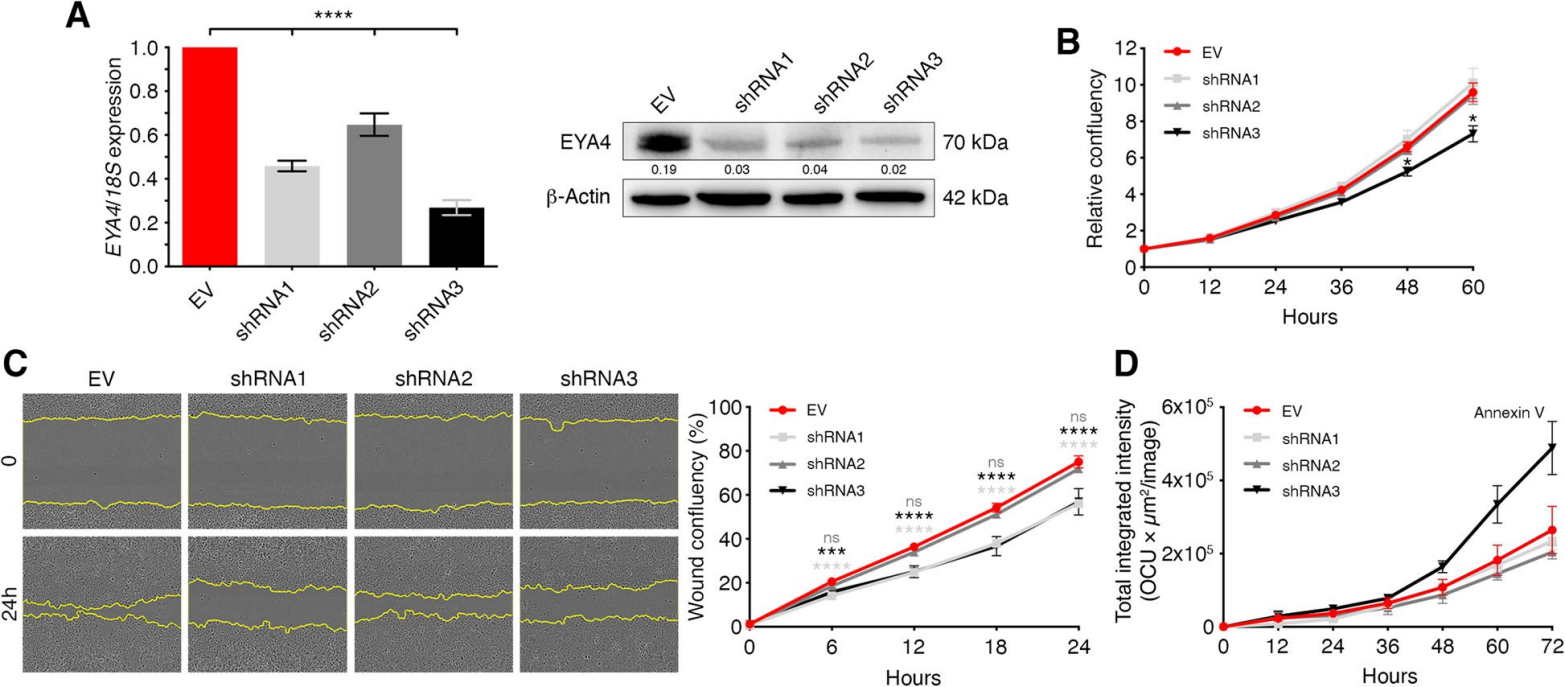
EYA4 regulates proliferation and migration in cells. **A** *EYA4* knockdowns in HeLa cells (mean ± SEM; 3 biological replicates). β-Actin was used as the loading control. Western blot was repeated at least three times. Quantification was done on the represented immunoblot. **B** Representative growth curves of HeLa control and EYA4 depleted cells. Proliferation was measured by live imaging. Data represent the mean relative confluency ± SEM of three independent experiments. **C** Representative images of an in vitro wound-healing assay monitored by live imaging and the corresponding quantification of the migration area after wounding are shown. Data represent the mean wound confluency ± SEM of three independent experiments. **D** Annexin V-labeled apoptotic HeLa cells were measured by live imaging. Data represent the mean total integrated intensity ± SEM of three independent experiments. For all panels * *P* ≤ 0.05, *** *P* ≤ 0.001, **** *P* ≤ 0.0001

One simple explanation for variations in primary tumor sizes is the accumulation of larger cells [33, 34] or increased proliferation rates. Uncontrolled and unlimited cell proliferation is a hallmark of cancer [35] and another member of the Eyes Absent family, EYA2, has been shown to increase cell proliferation in lung cancer [36]. We generated stable knockdowns in HeLa cells, using three independent short-hairpin RNAs, and a significant decrease in EYA4 protein levels was achieved (Fig. 3A). We followed growth rates by live-cell imaging. In both, HeLa (Fig. 3B) and MDA-MB-231 (Fig. S3A) cells, depletion of EYA4 led to lower proliferation rates compared to control. On the contrary, the over-expression of EYA4 in MCF-7 leads to higher proliferation rates when compared to control (Fig. S3C), suggesting that EYA4 promotes cell proliferation. In addition, we investigated the effect of EYA4 on cell migration by comparing the number of control, EYA4 knocked down and EYA4 over-expressing cells at the scratch wound at different time points by live-cell imaging. HeLa (Fig. 3C) and MDA-MB-231 (Fig. S3B) cells depleted for EYA4, exhibited significantly lower migratory capacity relative to cells expressing the empty vector (EV) control, whilst EYA4 over-expression in MCF-7 (Fig. S3D) primes the migration capacity of cells, indicating that EYA4 plays a role in driving cell migration. EYA4 phosphatase mutants, especially the S/T phosphatase deficient mutant, display a phenotype comparable, or even more dramatic, than EYA4 depleted cells when tested for proliferation and migration capacities in HeLa cells (Fig. S3E-J), showing a significant decrease for both. However, we did not observe the same phenotype in MDA-MD-231 cells (Fig. S3K-N), suggesting that the role in cell migration might be cell line dependent. As we cannot exclude that apparent slower proliferation is caused by cell death, we followed HeLa control and EYA4 knockdown cells after the addition of the apoptosis marker, annexin V. Compared to HeLa control cells, EYA4 shRNA3 showed a slight increase in apoptosis in normal growth conditions (Fig. 3D and Fig. S4A), which could explain, at least partially, the slower proliferation rate observed for shRNA3. The increase in apoptosis observed in HeLa EYA4 shRNA3 cells reflects the fact that this hairpin could not be used in MDA-MB-231 cells, as severe knockdown of EYA4 is incompatible with cell viability.

### EYA4 perturbs cell cycle progression

**Fig. 4:**
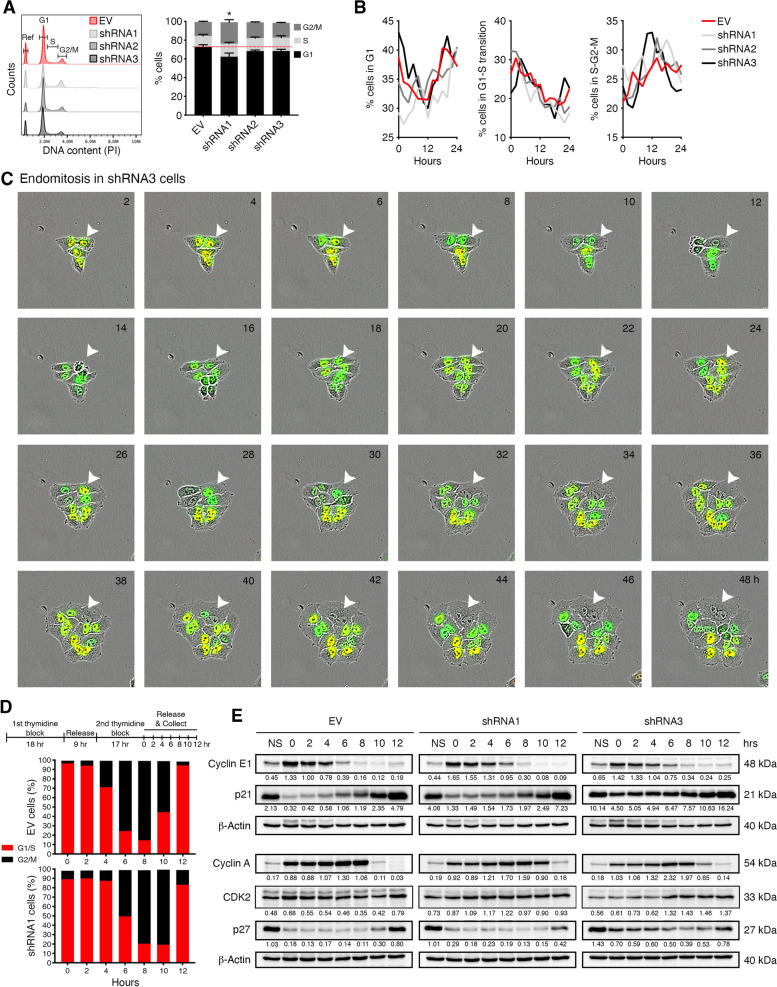
EYA4 disturbs cell cycle progression and triggers cell cycle checkpoints. **A** Analysis of cell cycle distribution of asynchronized populations by flow cytometry. A representative cell cycle histogram is shown. The percentage of cells in G1, S or G2/M is plotted as mean ± SEM for three independent experiments, * *P* ≤ 0.05. **B** Percentage of HeLa FUCCI cells, transduced with EV control or EYA4 shRNAs, expressing each marker classified as in G1, G1-S transition or S-G2-M is plotted. **C** Representative images showing endomitosis (marked by white arrowhead) in HeLa FUCCI EYA4 depleted cells. **D** A schematic representation of double thymidine block for cell synchronization in early S-phase is shown. Cell synchronization was monitored by flow cytometry of PI-stained cells. The percentage of cells in G1/S or G2/M is plotted for three independent experiments. **E** Immunoblotting of cell cycle checkpoints proteins (cyclin E, p21, cyclin A, CDK2, and p27) in EYA4 depleted cells and controls, after double thymidine block (NS, non-synchronized population). β-Actin was used as the loading control. Western blots were repeated at least three times. Quantification was done on the represented immunoblot

Cell cycle is tightly regulated via checkpoints that are activated by DNA damage, low nutrient content, or other endogenous and external stresses. Aberrant cell cycle progression tends to result in genome instability and contributes to cancer progression. To determine how EYA4 might affect cell cycle progression, flow cytometry was used to profile asynchronous populations of either control or EYA4-depleted cells (Fig. 4A). We observed a slight increase (2–3%) in S-phase when EYA4 is silenced and a significant increase (8%) in the G2/M population for shRNA1 (Fig. 4A), when compared to empty vector control, which suggested a delay in cell cycle progression upon EYA4 depletion. However, shRNA3 does not show a significant increase in G2/M, which could be explained due to its characteristic phenotype (enlarged, flat and multinucleated cells, Fig. S4B), and this subpopulation could have been gated out by flow cytometry (raw data in Fig. S5). We used the FUCCI system [20] and live-cell imaging (Fig. 4B and Fig. S6A-B) to overcome these technical issues and profile single cells. We observed a subtly different behavior for EYA4 shRNA3, especially when it comes to cells in S-G2-M (Fig. 4B). This correlates with cells depleted for EYA4 (especially with shRNA3) undergoing endoreplication (Fig. 4C). Endoreplication refers to a cell cycle variant that only consists of the G and S phases, during which cells replicate their DNA content without dividing, thus giving rise to polyploid cells [18, 37]. The result is either a cell that maintains separate nuclei and remains multinucleated, due to a process called endomitosis, or a cell with an enlarged-single nucleus containing all the DNA, derived from a process called endocycling [18]. As described above, shRNA3 cells tend to be enlarged and multinucleated, which is characteristic of endomitosis, a major form of endoreplication in which mitosis is initiated but not completed (green/non-fluorescent/green; white arrowhead; Fig. 4C). The endoreplication and consequent polyploidy observed, which can occur in response to stress, is a phenomenon that has been linked to cancer progression and chemotherapy resistance [38].

### EYA4 depletion induces cell cycle arrest

The most common cause of endoreplication cycling is a switch in activation/inactivation of cyclins and cyclin-dependent kinases (CDKs), key regulators of cell cycle progression [39]. To investigate if EYA4 expression impacts individual phases of the cell cycle, cells were arrested in early S-phase with a double thymidine block (Fig. 4D) and assessed for cell cycle progression. Propidium iodide (PI) staining of the DNA and flow cytometry in HeLa cells showed that EYA4 decrease (shRNA1) leads to a delay in S-phase restart compared to control (Fig. 4D). Upon release, 74.9% of control cells entered G2/M after 6 h, compared to 49.43% of EYA4-depleted cells (raw data can be found in Fig. S5). EYA4-depleted cells resumed/finished S with a 2 h delay, and 78.3% of depleted cells entered G2/M 8 h post-release, showing that EYA4 depletion extends S-phase and delays cell division. The most logical explanations for such observations are defects in DNA replication and aberrant checkpoint signals. Since EYA4 depletion appears to halt the cell cycle in the transition between S-phase and G2, we evaluated the activation of several proteins involved in G1 and G2 checkpoints (schematic in Fig. S6C). We first examined the G1/S transition to assess if the cells can initiate DNA replication, by determining the expression of cyclin E1, its partner CDK2, and its corresponding CDK inhibitors, p21^WAF1/CIP1^ and p27^KIP1^ (Fig. 4E). After double thymidine block that synchronizes cells in early S, EYA4 depleted cells appeared to accumulate p21^WAF1/CIP1^ and p27^KIP1^, especially when residual EYA4 expression is minimal, as with shRNA3. However, CDK2 does not seem to be affected by EYA4 depletion. Cyclin E1 levels increase sharply in late G1, where it interacts and activates CDK2 allowing G1/S transition, then decrease in S-phase [40]. In EYA4-depleted cells, accumulation of Ccyclin E1 is minimal despite the synchronization but remains elevated up to 6 h. This correlates with the accumulation of cells in G1-S at 6 h observed in cells depleted for EYA4 (Fig. 4D). Cyclin E1/CDK2 is an important part of the G1 checkpoint and deregulation in the G1/S transition may impair normal DNA replication, causing replication stress and DNA damage [41]. Upon release from the thymidine block, we observed that EYA4-depleted cells exhibit a notable delay in S-phase compared to the EV control (Fig. 4D). This was confirmed by the accumulation of cyclin A (highly expressed in S-phase, decreasing in G2) for up to 10 h post-release (Fig. 4E). Altogether, these data indicate that in the absence of EYA4, S-phase and its subsequent transition into G2 become prolonged. These effects could stem from faulty DNA replication and/or the accumulation of DNA damage during S-phase.

### Spontaneous replication stress is observed in the absence of EYA4

**Fig. 5:**
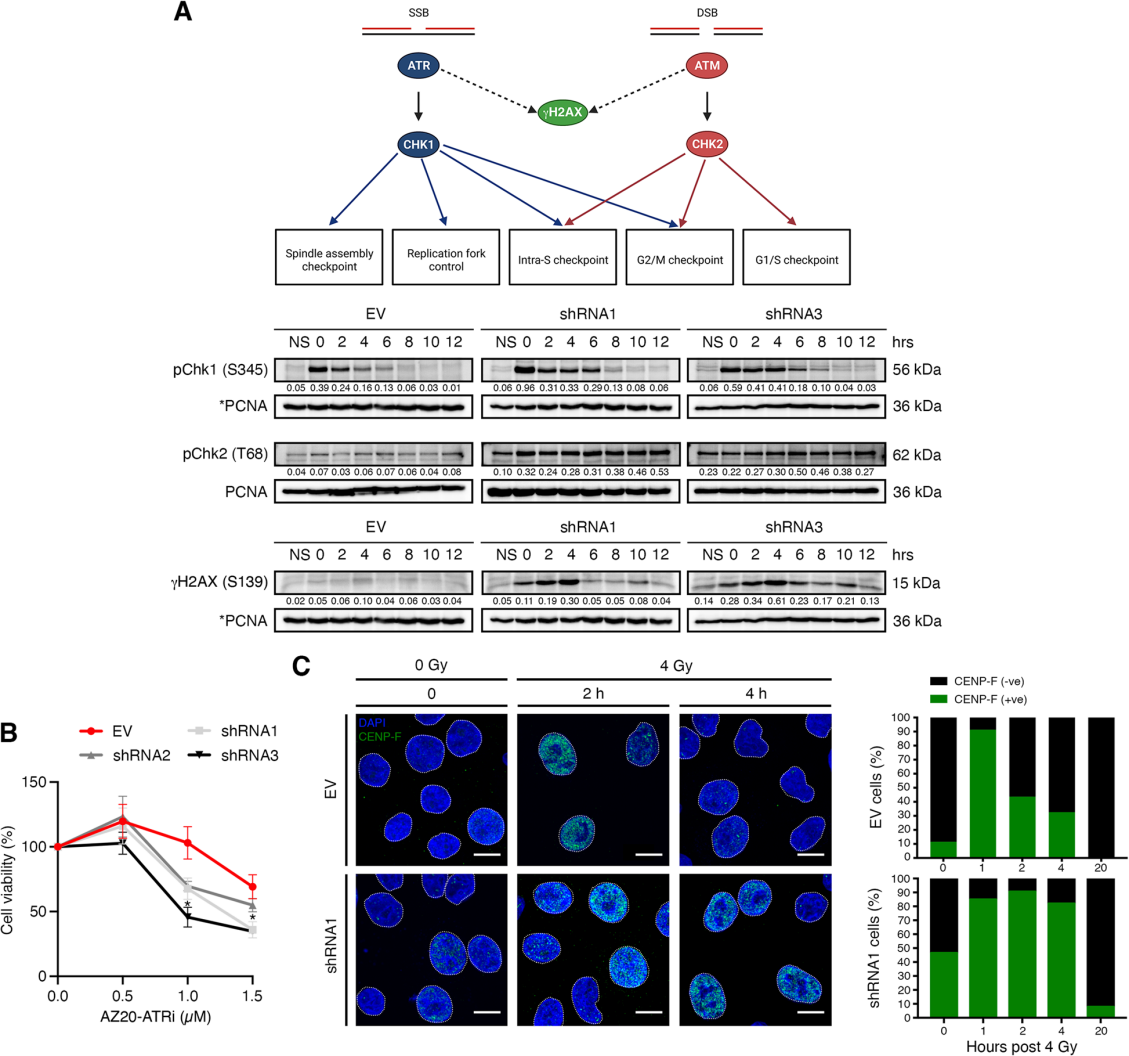
EYA4-depleted cells accumulate spontaneous replication stress. **A** Chk1 and Chk2 kinases in checkpoint control. A schematic representation of cell cycle checkpoints is shown (adapted from Collins and Garrett [44]). After double thymidine block, cells were released in early S-phase, activation of checkpoint kinase 1 and 2 (S345 and T68, respectively) and accumulation of spontaneous double-strand breaks in S-phase marked by γH2AX were followed by immunoblotting (NS, non-synchronized population). PCNA was used as the loading control. *Antibodies screened on the same membrane. Western blots were repeated at least three times. Quantification was done on the represented immunoblot. **B** EYA4-depleted cells show sensitivity to an ATR inhibitor, AZ20, using an MTT assay (mean ± SEM; *n* = 3). **C** Induction of CENP-F foci formation after exposure to γ-irradiation (4 Gy). Representative images (scale bar 10 μm) and quantification (n ≥ 100) in controls and cells depleted for EYA4 are shown

EYA4-depleted cells transition through G1/S and enter DNA replication but S-phase is extended and the S-G2 transition halted. We decided to evaluate the level of expression of pChk1 (S345) and pChk2 (T68) by immunoblotting, to assess if the cells accumulate spontaneous damaged DNA in the absence of EYA4. To do so, cells were arrested in early S-phase with a double thymidine block. Checkpoint kinase 1 (Chk1) is a key player of DNA-damage-activated checkpoint response that acts downstream of ATR (Ataxia Telangiectasia and Rad3 related) kinase, in response to the formation of single-stranded DNA due to DNA damage of blocked replication forks (Fig. 5A). EYA4-depleted cells accumulated pChk1 (S345) up to 8 h after thymidine-block release (Fig. 5A), but not the control, implying that replication fork stalling occurs in the absence of EYA4, and replication forks are not restarted. Chk2 is a stable protein expressed throughout the cell cycle. In response to DNA double-strand breaks, Chk2 becomes rapidly phosphorylated at threonine 68 by ATM (Ataxia Telangiectasia Mutated) (Fig. 5A). The kinase activity of Chk2 depends on the severity of DNA damage [42]. We found elevated pChk2 (T68) in the absence of EYA4 (Fig. 5A), which suggests the accumulation of double-stranded breaks (DSBs) that might be a consequence of replication fork collapse. Spontaneous accumulation of DNA damage was confirmed by evaluating the expression of the phosphorylated histone variant H2AX (S319, γH2AX) in early S-phase. Accumulation of γH2AX was observed in EYA4-depleted cells (Fig. 5A), but not in the control, consistent with the presence of replication stress suggested by pChk1 and pChk2 accumulation (Fig. 5A). In accordance with these results, cells depleted for EYA4 were also found sensitive to AZ20, an ATR inhibitor (Fig. 5B). We next sought to assess if the cells are able to progress throughout the cell cycle upon DNA damage induction. We followed the formation of CENP-F foci after 4 Gy γ-irradiation to identify cells in S-phase and G2/M. CENP-F gradually accumulates during the cell cycle until it reaches peak levels in G2/M phases [43]. Control cells accumulate in S-G2/M after γ-irradiation, indicating that the cell cycle is halted (1 h after 4 Gy) but they progress once DNA damage is resolved. Nevertheless, in the absence of EYA4, accumulation of CENP-F was observed up to 4 h after irradiation, indicating that the cells are taking longer to resolve DNA damage (Fig. 5C), and that the cell cycle is halted in G2/M, preventing cell division.

### EYA4 contributes to HU resistance

**Fig. 6:**
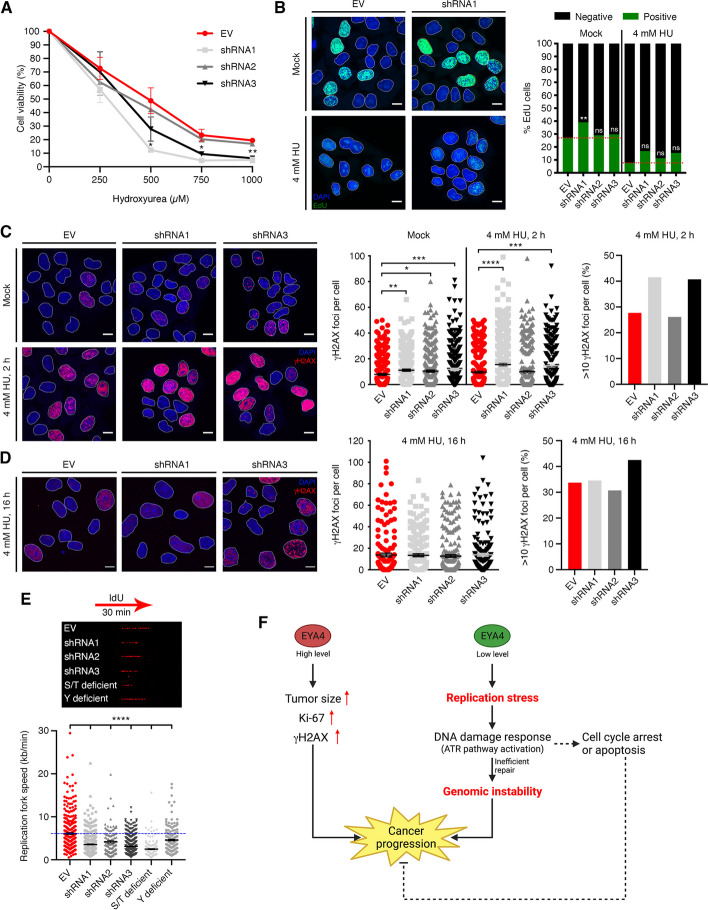
The impact of EYA4 on replication fork progression. **A** EYA4-depleted cells show sensitivity to hydroxyurea using an MTT assay (mean ± SEM; *n* = 3). **B** DNA synthesis was assessed by EdU incorporation. Representative images (scale bar 10 μm) and percentage of EdU incorporation in the presence of 4 mM hydroxyurea are shown for three independent experiments. **C**-**D** Accumulation of hydroxyurea-induced γH2AX foci formation. Representative images (scale bar 10 μm), quantification (mean ± SEM; n ≥ 350), and more than 10 foci per nucleus are shown for 4 mM HU treatment for 2 h followed by 2 h release (**C**) or 16 h HU treatment followed by 18 h release (**D**). **E** A representative DNA fiber image is shown for each genetic condition. Replication fork speed (kb/min) is shown for empty vector control, EYA4-depleted cells and EYA4 phosphatase mutant cells (mean ± SEM; a minimum of 150 forks was scored in two independent experiments yielding similar results. Statistical analysis: unpaired *t*-test). For all panels * *P* ≤ 0.05, ** *P* ≤ 0.01, *** *P* ≤ 0.001, **** *P* ≤ 0.0001. **F** Model: Over-expression of EYA4 leads to an aggressive and invasive breast cancer phenotype. EYA4 has a protective role in cells against replication stress, triggering the activation of the ATR pathway and cell cycle arrest

To address the potential role of EYA4 in the cellular response to replication stress, we examined the effects of knocking down EYA4 on the sensitivity to hydroxyurea (HU), which causes replication stress by depleting the intracellular pool of deoxynucleotides [45]. In accordance with the accumulation of replication stress and checkpoint activation, cells depleted for EYA4 were found to be sensitive to hydroxyurea in an MTT assay (Fig. 6A). In order to monitor DNA synthesis, we treated cells with 4 mM hydroxyurea for 2 h and then measure 5-ethynyl-2’-deoxyuridine (EdU) incorporation after the removal of HU. Under these conditions, silencing EYA4 resulted in a slightly increased rate of EdU incorporation (Fig. 6B), indicating that EYA4 might be involved in maintaining replication fork stability since EYA4-depleted cells appear to overcome the HU blockage and resume synthesis.

### EYA4 depletion results in increased and unresolved levels of HU-induced DSBs

Replication fork collapse resulting from chronic HU exposure generates double-stranded breaks [45], which are rapidly marked by γH2AX. To examine the possible role of EYA4 in the repair of HU-induced DSBs, HeLa cells were incubated with 4 mM HU for 2 h and then allowed to recover for 2 h in the absence of the drug. Even though EYA4-depleted cells have high levels of endogenous DNA damage, an increase in HU-induced DSBs was observed in the absence of EYA4 (Fig. 6C). Next, HeLa cells were incubated with 4 mM HU for 16 h and then released for 18 h, to assess for unresolved DNA damage in the absence of EYA4. Although residual γH2AX foci were present in HeLa control cells after recovery from HU exposure, ~ 10% more cells with > 10 γH2AX foci per cell (Fig. 6D) were observed in the absence of EYA4, implying that these cells have a diminished ability to resolve HU-induced DNA damage. Together, our results suggest that EYA4 contributes to replication-associated DNA damage repair.

### EYA4 impacts replication fork speed

To investigate the functional role of EYA4 in DNA replication, we utilized single-molecule analysis of replicated DNA fibers to test if the increased DSBs in EYA4-deficient cells affected replication fork (RF) progression and speed. We found that the replication tracts are much shorter in EYA4 deficient cells compared to control cells, under normal conditions (Fig. 6E), demonstrating that genome-wide RF progression is strongly impaired by EYA4 depletion. Interestingly, the fork slowing observed was even more dramatic in the S/T phosphatase deficient mutant cells, but not in the Y phosphatase deficient mutant cells, showing that the serine/threonine phosphatase activity of EYA4 is essential for replication fork progression.

## Discussion

EYA protein phosphatases have been associated with cancer pathologies, and they exhibit characteristics of oncogenic and tumor-suppressive activities depending on the tissue of origin. Because EYA are protein phosphatases, it is expected that lack of phosphorylation caused by their over-expression would impact a variety of cellular pathways in a tissue-specific manner, depending on the protein substrates expressed and targeted by EYA. Even though EYA proteins possess similar attributes in function, substrates, and carcinogenic power, only about 40% of the protein sequence is conserved between EYA1, 2, 3 and EYA4. Therefore, in this study, we sought to gain a better understanding of the cellular processes impacted by *EYA4* over-expression in cancer, and specifically understand the possible role of EYA4 in promoting and sustaining carcinogenesis of the breast.

Together with collaborators, we have reported that the *EYA4* gene is hypermethylated in the first intron–exon junction [15], and possibly over-expressed in triple-negative breast cancer patients, which correlates with publicly available TCGA dataset that shows amplification as the most common alteration in breast cancer patients. It is generally accepted that DNA methylation most frequently leads to repression of gene expression. However, the consequences of methylation for gene expression can be more complex as shown in recent years by genome-wide methylation analyses. Studies have revealed that DNA methylation can lead to the activation or repression of gene expression based on the sites of DNA methylation and on the density of non-core histones deposition [46, 47]. Rauscher, et al*.* [46] found that all the promoter regions that displayed invasive breast cancer-linked hypermethylation exhibited an inverse correlation with gene expression. However, they found regions that displayed a positive correlation between methylation and gene expression, and these included far-upstream and intragenic regions, which is consistent with our notion of a positive correlation between EYA4 methylation and gene expression. Ultimately, this requires further investigation in order to understand the consequences of gene expression, and in the present study, we found that EYA4 is significantly over-expressed in breast carcinoma compared to normal breast tissue, using immunohistochemistry analyses.

In this study, we used HeLa and breast cancer cell lines to investigate the proliferation rates of cells knocked down or over-expressing EYA4, ex vivo and as xenografts in small animals. While EYA4 is often not expressed in normal breast, we found that MDA-MB-231 over-express EYA4, and are depending on its expression for survival. We show that over-expression of EYA4 drives the growth of ER^+^ primary tumors, and promotes metastasis to distant organs such as lungs and livers. In triple-negative breast cancer xenografts, the knockdown of EYA4 was able to efficiently limit the spread of metastasis and the overall cancer burden. These two xenograft studies suggest that EYA4 therapeutic targeting is an interesting avenue that should be pursued for anti-breast cancer drug development. Over-expression of EYA4 in cancer could be used to predict patient outcomes and drug response.

EYA4 level is inversely correlated with ER status, with high expression largely found in triple-negative breast cancer, while ER^+^ tumors and cell lines express little or no EYA4. This warrants further investigation to fully understand the connection between EYA phosphatases and the hormonal status of cancerous tissues. In breast cancer, it is well-established that estrogen is a major driver of breast tumor growth through its role in cell proliferation, as well as an effective therapeutic target. It has been proposed that in MCF-7 cells, ER-α induces cell proliferation by regulating the cell cycle by stimulating the expression of PCNA and Ki-67 and suppressing of p53/p21 transcription [31]. Our data shows that EYA4-depleted cells exhibit slower division rates as measured by live imaging. Further investigation by live imaging and the FUCCI system demonstrated that cells silenced for EYA4 undergo slower DNA synthesis, halting cell cycle progression, and undergoing endoreplication as a result of missed mitosis initiation. Our data are in line with previous observations in glioma where EYA4 over-expression promotes cell proliferation by directly suppressing the expression of p27^KIP1^, suggesting p27^KIP1^ as a transcriptional target of EYA4 [48].

In EYA4-depleted cells, we observed cell cycle arrest and DNA damage response (DDR) activation. We have shown that EYA4, and specifically the serine/threonine domain of EYA4, plays an important novel role in replication fork progression. Several human diseases have been associated with defects in replication stress signaling, including Bloom syndrome [49], Fanconi anemia [50], Seckel syndrome [51], Werner syndrome [52], and the most common one, cancer [53]. To our knowledge, this is the first evidence implicating EYA4, or any member of the EYA family, in the resolution of DNA replication-induced DNA damage. These results highlight the need for further characterization of the roles of EYA proteins in the DDR and genomic integrity.

Importantly, we have shown that the serine/threonine phosphatase activity of EYA4 is important for breast cancer progression and metastasis, suggesting that targeting the EYA4 phosphatase activity could help devise new cancer treatments directed against primary tumors and distant metastasis.

## Conclusions

This is the first study to explore the role of EYA4 in replication-induced DNA damage repair. EYA4 is an important novel breast cancer gene and prognostic marker, with the potential to be a valuable diagnostic and therapeutic target for triple-negative breast cancer.

### Supplementary Information

**Additional file 1: Supplemental Figure S1. **Expression pattern of eyes absent 4 (EYA4) in breast cancer. (A) EYA4 contains a serine/threonine phosphatase domain (residues 268-292) and a tyrosine phosphatase domain, which is comprised of 4 individual motifs (covering residues 369-614). Mutations utilized in this study are indicated by stars (red = S/T phosphatase deficient and green = Y phosphatase deficient). (B-C) EYA4 expression in different breast cancer cell lines (mean ± SD; *n*=3). GAPDH was used as the loading control. Quantification was done on the represented immunoblot. (D) Normal breast tissues (*n*=3; 1 replicate) and breast carcinoma tissues (*n*=12; 2 replicates) were stained by the Human Protein Atlas (HPA) as described [22], using DAB-labeled antibody HPA038771. Numbers refer to patient IDs. For clinical details corresponding to these patient IDs, refer to Table S2. (E) Immunohistochemistry-based H-scores between lobular (*n*=3) and ductal (*n*=9) breast carcinomas. Error bars show means ± SEM. *P* value: Student t-test with Welch’s correction. (F) EYA4 knockdowns in MDA-MB-231 cells (mean ± SEM; 3 biological replicates). (G) EYA4 over-expression in MCF-7 cells (mean ± SEM; 3 biological replicates). For F and G, **** *P* ≤ 0.0001. **Supplemental Figure S2.** The serine/threonine phosphatase domain of EYA4 is essential for breast cancer progression. (A-D) Analysis of lungs after mouse lateral tail-vein injections. (A and C) Representative images of surgically removed lungs (scale bar 5 mm) are shown. (B and D) Hematoxylin and eosin staining of lungs dissected 4-5 weeks post tail-vein injection. Low magnification (scale bar 5 mm), and high magnification views (scale bar 50 µm). Black lines indicate the areas shown at higher magnification. (E-F) Representative images of surgically removed and immunohistochemistry-stained (E) lungs and (F) livers are shown. Low magnification (scale bar 2 mm), mid magnification (scale bar 200 µm), and high magnification views (scale bar 50 µm). Black boxes indicate the areas shown at higher magnification. **Supplemental Figure S3.** EYA4 regulates proliferation and migration in cells. (A-D) Proliferation (A and C) and migration (B and D) were monitored by live imaging in EYA4-depleted MDA-MB-231 cells (A-B) and over-expressed MCF-7 cells (C-D). Data represent the mean ± SEM of three independent experiments. (E-N) Proliferation and migration were monitored in EYA4 phosphatase mutant cells, in two different cell lines (E-J HeLa; and K-N MDA-MB-231/Luc). Data represent the mean ± SEM of three independent experiments for F, G, H, J, L and N. For MTT assay shown in I and M, data represents the median (n=10 for I and n=24 for M). For all panels * *P* ≤ 0.05, ** *P* ≤ 0.01, *** *P* ≤ 0.001, **** *P* ≤ 0.0001. **Supplemental Figure S4. **EYA4-depleted cells characteristic phenotype. (A) Representative images of annexin V-labeled apoptotic HeLa cells monitored by live imaging. (B) Representative images of EYA4 knockdown phenotype (enlarged, flat and multinucleated cells). **Supplemental Figure S5. **Cell cycle analysis using FlowJo. HeLa control and EYA4 knockdown cells were synchronized in early S-phase and monitored at T=0, 2, 4, 6, 8, 10 and 12 hours. NS, non-synchronized population. Trout erythrocytes were used as an internal DNA control reference, to normalize the cell cycle and be able to follow cell cycle progression. Cell synchronization was monitored by flow cytometry of propidium iodide-stained cells. Three biological replicates are shown. **Supplemental Figure S6.** EYA4 cell cycle progression monitored by live imaging utilizing the FUCCI system. (A) Schematic representation of the FUCCI system, adapted from Sakaue-Sawano, et al. [20], created with BioRender. During G1 phase, the nuclei of FUCCI-expressing cells appear red due to stabilization of mKO2-hCdt1 and ubiquitin-dependent proteolysis of mAG-hGem. As cells transition from G1 to S phase, both (mKO2-hCdt1(+)/mAG-hGem(+)) are stabilized to different degrees, resulting in nuclei with a yellowish shade. Once the cells have transitioned to S-phase, mAG-hGem is stabilized and mKO2-hCdt1 is degraded, causing the nuclei to appear green, which is maintained throughout S, G2 and M phases. For a brief period of time, during M to G1 transition, fluorescence signal is lost due to the simultaneous degradation of both probes. (B) Representative images of HeLa FUCCI control and EYA4 depleted cells monitored by live imaging. (C) Schematic representation of cell cycle regulation, created with BioRender.

**Additional file 2: Supplemental Table S1. **Immunohistochemistry analysis of EYA4 expression in breast cancer [22]. **Supplemental Table S2.** Clinical details and IHC H-scores for breast tissues^a^.

## Data Availability

All materials underlying this study are available upon request. All data generated or analyzed during this study are included in the main text or the supplementary information.

## Abbreviations

ATM: Ataxia Telangiectasia Mutated
ATR: Ataxia Telangiectasia and Rad3 related
BLI: Bioluminescence Intensity
CCD: Charged-Coupled Device
CDK: Cyclin-Dependent Kinase
Chk1: Checkpoint kinase 1
Chk2: Checkpoint kinase 2
DDR: DNA damage response
DSB: Double-stranded break
ED: EYA domain
EdU: 5-Ethynyl-2’-deoxyuridine
ER: Estrogen Receptor
EV: Empty Vector
EYA: Eyes Absent
FL: Full Length
FUCCI: Fluorescent, Ubiquitination-based Cell-Cycle Indicators
HAD: Haloacid Dehalogenase
HU: Hydroxyurea
IdU: 5-Iodo-2’-deoxyuridine
IHC: Immunohistochemistry
MFP: Mammary Fat Pad
MPNST: Malignant Peripheral Nerve Sheath Tumors
PI: Propidium Iodide
PR: Progesterone Receptor
RF: Replication Fork
ROS: Reactive Oxygen Species
shRNA: Short hairpin RNA
S/T: Serine/Threonine
WT: Wild Type
Y: Tyrosine
