# Supplementary material for: EYA4 promotes breast cancer progression and metastasis through its role in replication stress avoidance
Source: Mol Cancer. 2023 Sep 30;22:158. doi: 10.1186/s12943-023-01861-4 (PMC10543271; doi:10.1186/s12943-023-01861-4)
Supplement: Supplementary file 1 — Additional file 1: Supplemental Figure S1. Expression pattern of eyes absent 4 (EYA4) in breast cancer. (A) EYA4 contains a serine/threonine phosphatase domain (residues 268-292) and a tyrosine phosphatase domain, which is comprised of 4 individual motifs (covering residues 369-614). Mutations utilized in this study are indicated by stars (red = S/T phosphatase deficient and green = Y phosphatase deficient). (B-C) EYA4 expression in different breast cancer cell lines (mean ± SD; n=3). GAPDH was used as the loading control. Quantification was done on the represented immunoblot. (D) Normal breast tissues (n=3; 1 replicate) and breast carcinoma tissues (n=12; 2 replicates) were stained by the Human Protein Atlas (HPA) as described [22], using DAB-labeled antibody HPA038771. Numbers refer to patient IDs. For clinical details corresponding to these patient IDs, refer to Table S2. (E) Immunohistochemistry-based H-scores between lobular (n=3) and ductal (n=9) breast carcinomas. Error bars show means ± SEM. P value: Student t-test with Welch’s correction. (F) EYA4 knockdowns in MDA-MB-231 cells (mean ± SEM; 3 biological replicates). (G) EYA4 over-expression in MCF-7 cells (mean ± SEM; 3 biological replicates). For F and G, **** P ≤ 0.0001. Supplemental Figure S2. The serine/threonine phosphatase domain of EYA4 is essential for breast cancer progression. (A-D) Analysis of lungs after mouse lateral tail-vein injections. (A and C) Representative images of surgically removed lungs (scale bar 5 mm) are shown. (B and D) Hematoxylin and eosin staining of lungs dissected 4-5 weeks post tail-vein injection. Low magnification (scale bar 5 mm), and high magnification views (scale bar 50 µm). Black lines indicate the areas shown at higher magnification. (E-F) Representative images of surgically removed and immunohistochemistry-stained (E) lungs and (F) livers are shown. Low magnification (scale bar 2 mm), mid magnification (scale bar 200 µm), and high magnification views (scale bar 50 [file 12943_2023_1861_MOESM1_ESM.docx]

**Supplemental Figures and Figure Legends**

**
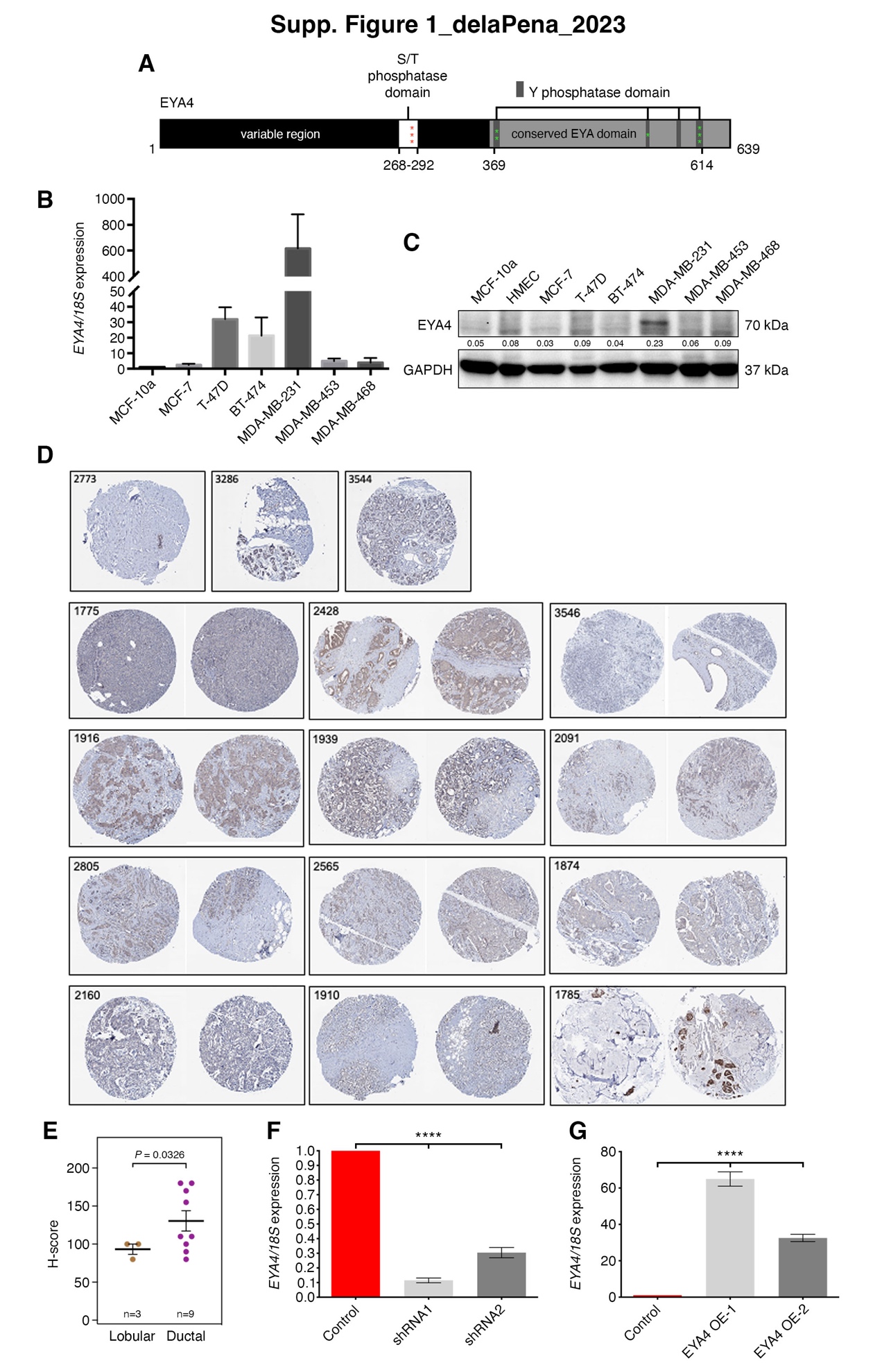
**

**Supplemental Figure S1: Expression pattern of eyes absent 4 (EYA4) in breast cancer.** (A) EYA4 contains a serine/threonine phosphatase domain (residues 268-292) and a tyrosine phosphatase domain, which is comprised of 4 individual motifs (covering residues 369-614). Mutations utilized in this study are indicated by stars (red = S/T phosphatase deficient and green = Y phosphatase deficient). (B-C) EYA4 expression in different breast cancer cell lines (mean ± SD; n=3). GAPDH was used as the loading control. Quantification was done on the represented immunoblot. (D) Normal breast tissues (n=3; 1 replicate) and breast carcinoma tissues (n=12; 2 replicates) were stained by the Human Protein Atlas (HPA) as described (22), using DAB-labeled antibody HPA038771. Numbers refer to patient IDs. For clinical details corresponding to these patient IDs, refer to Table S2. (E) Immunohistochemistry-based H-scores between lobular (n=3) and ductal (n=9) breast carcinomas. Error bars show means ± SEM. *P* value: Student *t*-test with Welch’s correction. (F) *EYA4* knockdowns in MDA-MB-231 cells (mean ± SEM; 3 biological replicates). (G) *EYA4* over-expression in MCF-7 cells (mean ± SEM; 3 biological replicates). For F and G, **** *P* ≤ 0.0001.

**
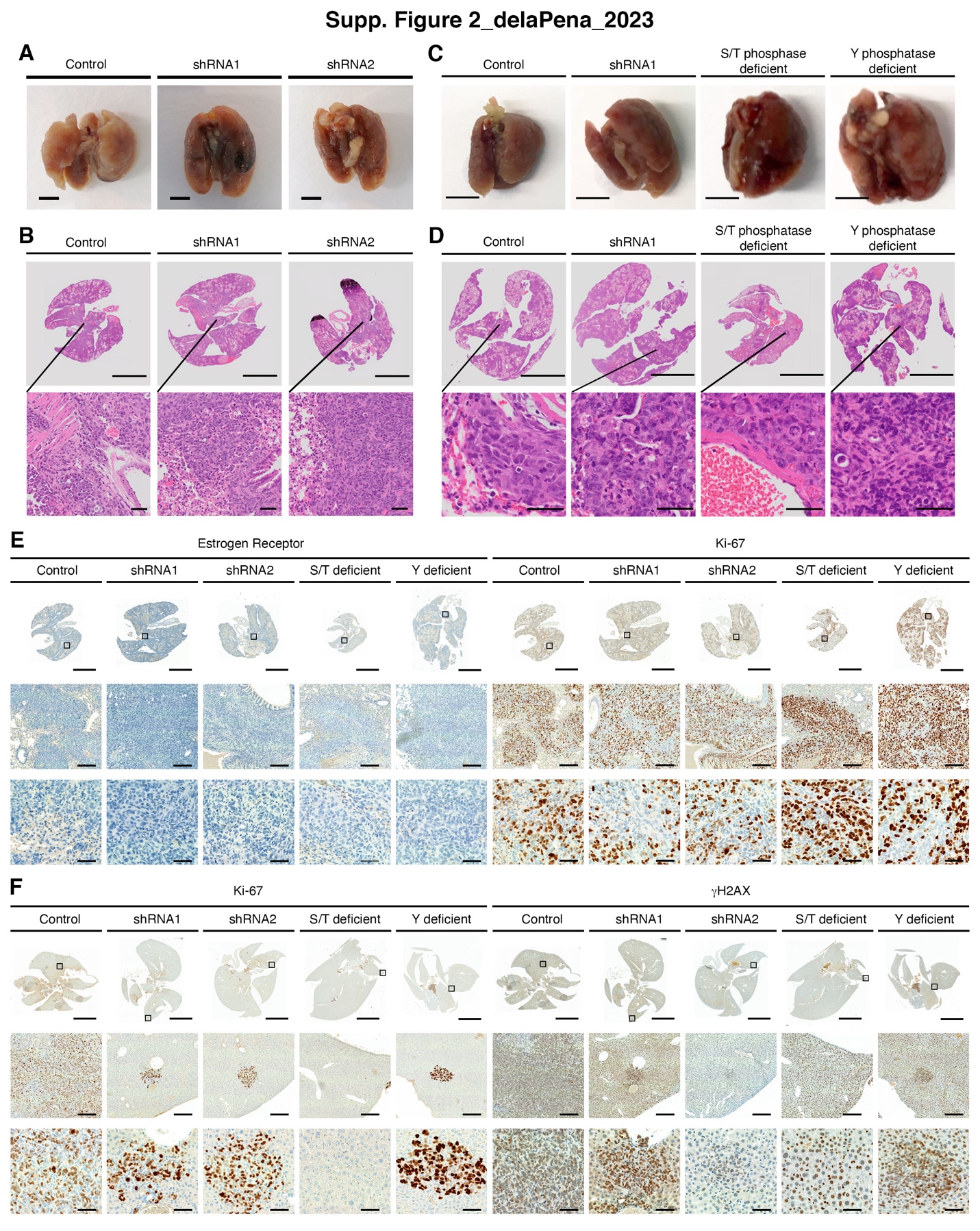
**

**Supplemental Figure S2: The serine/threonine phosphatase domain of EYA4 is essential for breast cancer progression.** (A-D) Analysis of lungs after mouse lateral tail-vein injections. (A and C) Representative images of surgically removed lungs (scale bar 5 mm) are shown. (B and D) Hematoxylin and eosin staining of lungs dissected 4-5 weeks post tail-vein injection. Low magnification (scale bar 5 mm), and high magnification views (scale bar 50 μm). Black lines indicate the areas shown at higher magnification. (E-F) Representative images of surgically removed and immunohistochemistry-stained (E) lungs and (F) livers are shown. Low magnification (scale bar 2 mm), mid magnification (scale bar 200 μm), and high magnification views (scale bar 50 μm). Black boxes indicate the areas shown at higher magnification.

**
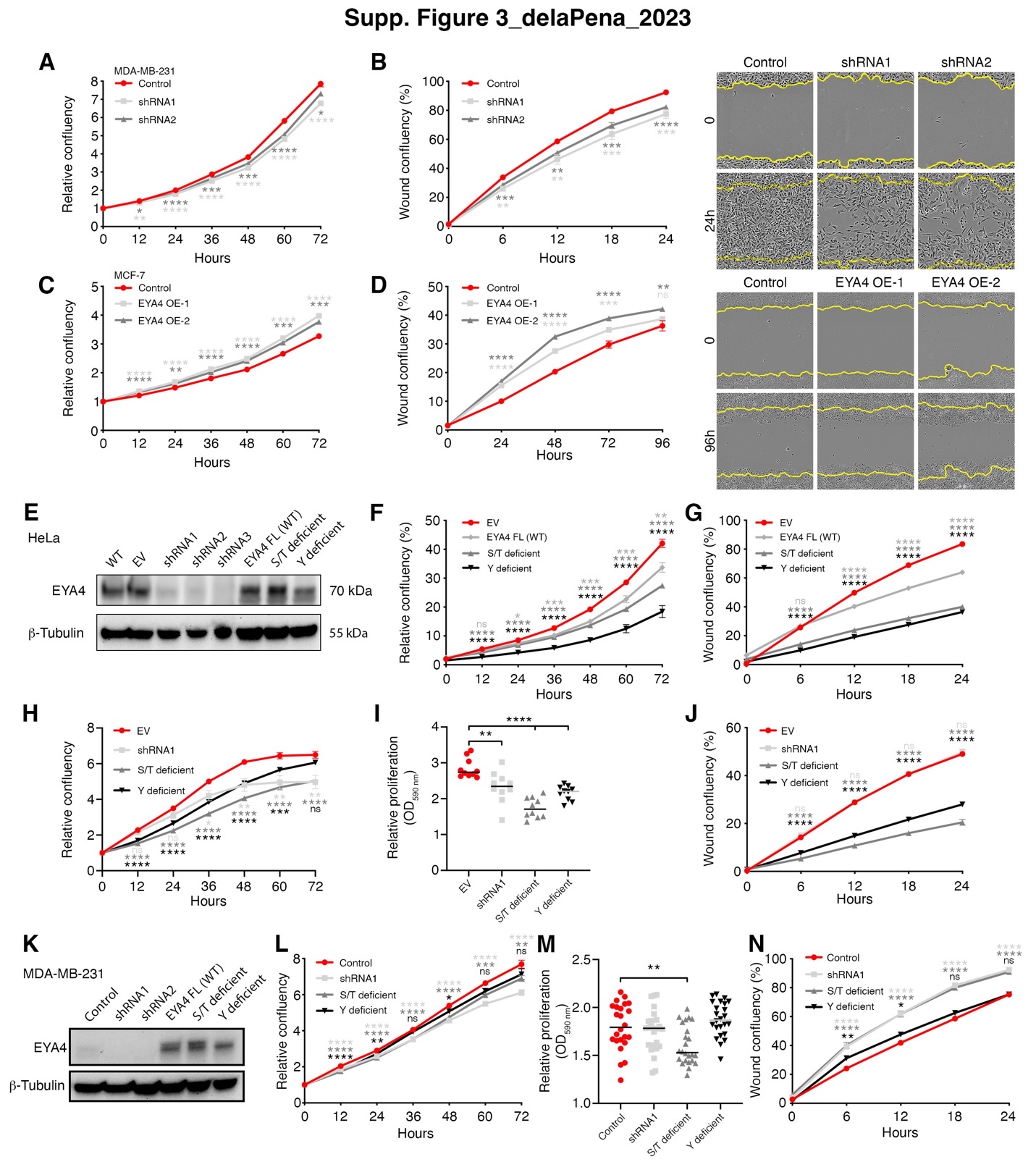
**

**Supplemental Figure S3: EYA4 regulates proliferation and migration in cells.** (A-D) Proliferation (A and C) and migration (B and D) were monitored by live imaging in EYA4-depleted MDA-MB-231 cells (A-B) and over-expressed MCF-7 cells (C-D). Data represent the mean ± SEM of three independent experiments. (E-N) Proliferation and migration were monitored in EYA4 phosphatase mutant cells, in two different cell lines (E-J HeLa; and K-N MDA-MB-231/Luc). Data represent the mean ± SEM of three independent experiments for F, G, H, J, L and N. For MTT assay shown in I and M, data represents the median (n=10 for I and n=24 for M).

**
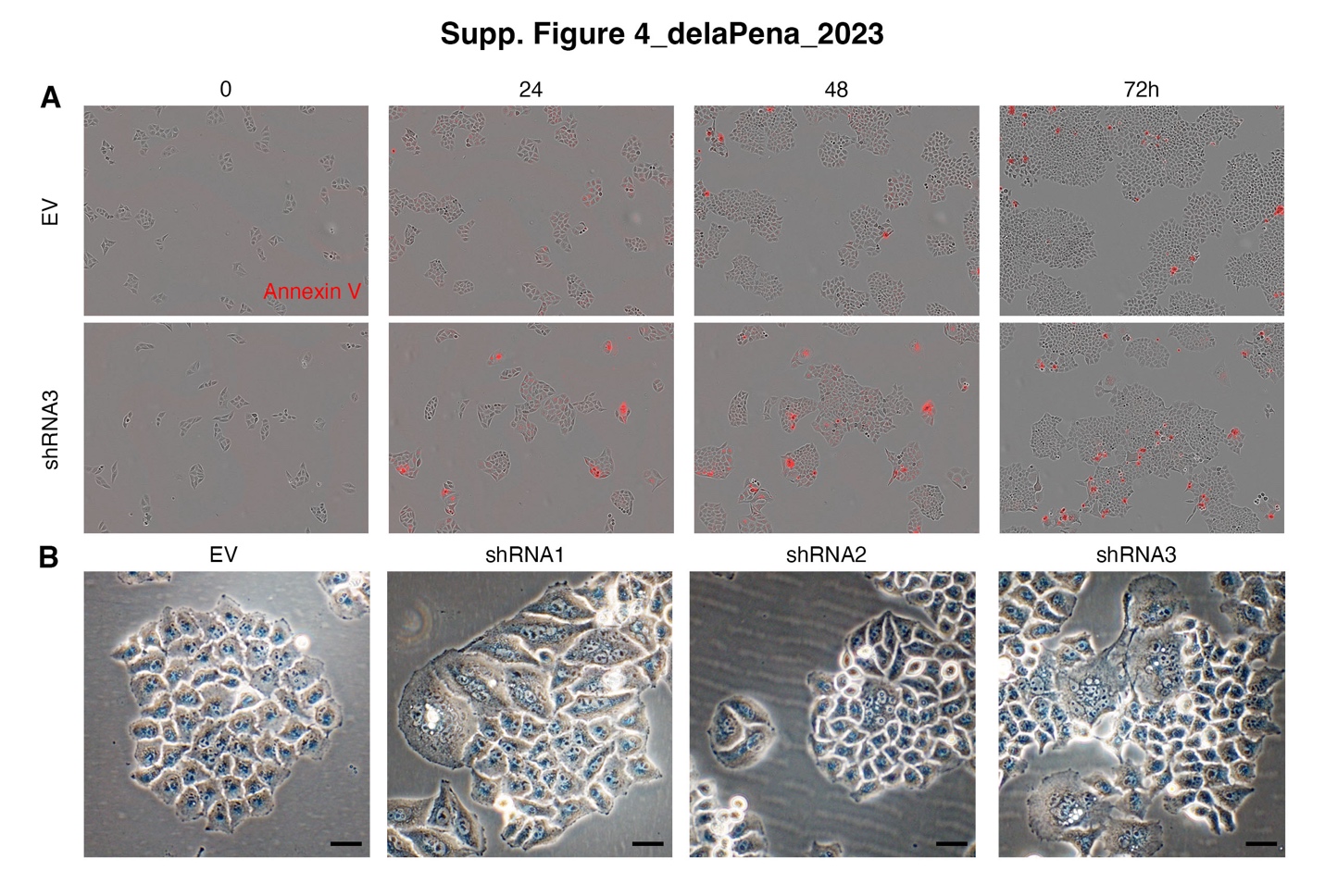
**

**Supplemental Figure S4: EYA4-depleted cells characteristic phenotype.**

(A) Representative images of annexin V-labeled apoptotic HeLa cells monitored by live imaging. (B) Representative images of EYA4 knockdown phenotype (enlarged, flat and multinucleated cells). For all panels * *P* ≤ 0.05, ** *P* ≤ 0.01, *** *P* ≤ 0.001, **** *P* ≤ 0.0001.


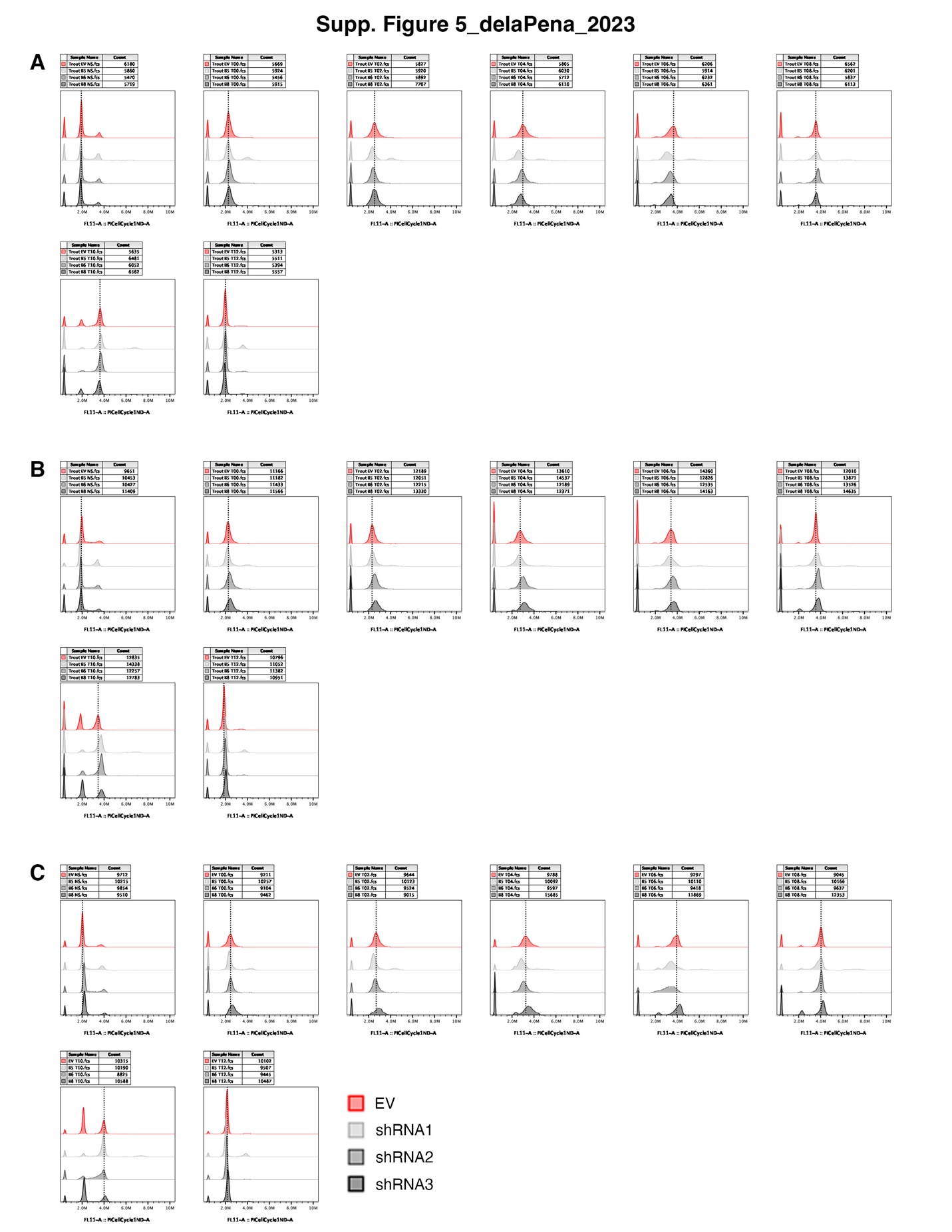


**Supplemental Figure S5: Cell cycle analysis using FlowJo.** HeLa control and EYA4 knockdown cells were synchronized in early S-phase and monitored at T=0, 2, 4, 6, 8, 10 and 12 hours. NS, non-synchronized population. Trout erythrocytes were used as an internal DNA control reference, to normalize the cell cycle and be able to follow cell cycle progression. Cell synchronization was monitored by flow cytometry of propidium iodide-stained cells. Three biological replicates are shown.


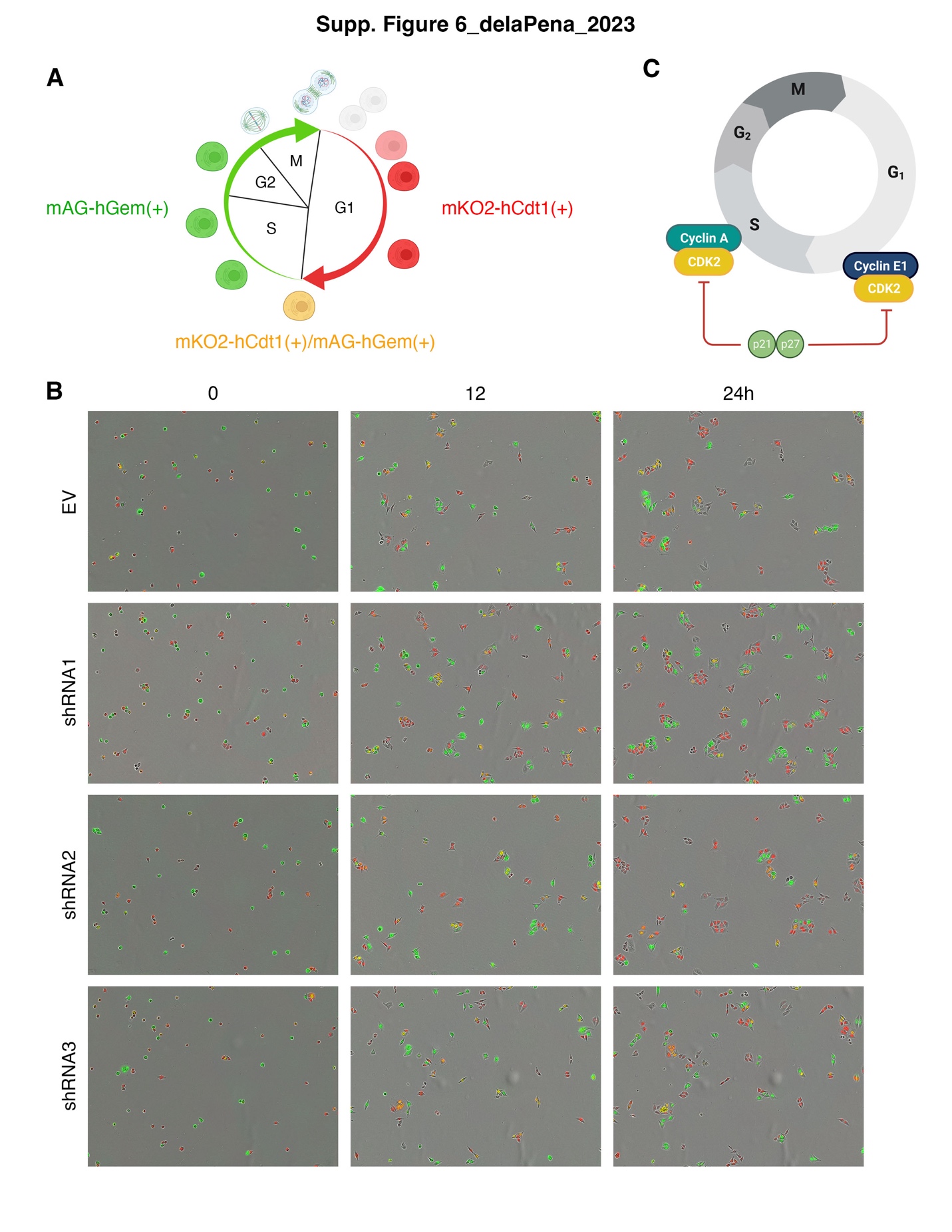


**Supplemental Figure S6: EYA4 cell cycle progression monitored by live imaging utilizing the FUCCI system*.*** (A) Schematic representation of the FUCCI system, adapted from Sakaue-Sawano, *et al.* (20), created with BioRender. During G1 phase, the nuclei of FUCCI-expressing cells appear red due to stabilization of mKO2-hCdt1 and ubiquitin-dependent proteolysis of mAG-hGem. As cells transition from G1 to S phase, both (mKO2-hCdt1(+)/mAG-hGem(+)) are stabilized to different degrees, resulting in nuclei with a yellowish shade. Once the cells have transitioned to S-phase, mAG-hGem is stabilized and mKO2-hCdt1 is degraded, causing the nuclei to appear green, which is maintained throughout S, G2 and M phases. For a brief period of time, during M to G1 transition, fluorescence signal is lost due to the simultaneous degradation of both probes. (B) Representative images of HeLa FUCCI control and EYA4 depleted cells monitored by live imaging. (C) Schematic representation of cell cycle regulation, created with BioRender.
