# Supplementary material for: EYA4 promotes breast cancer progression and metastasis through its role in replication stress avoidance
Source: Mol Cancer. 2023 Sep 30;22:158. doi: 10.1186/s12943-023-01861-4 (PMC10543271; doi:10.1186/s12943-023-01861-4)
Supplement: Supplementary file 2 — Additional file 2: Supplemental Table S1. Immunohistochemistry analysis of EYA4 expression in breast cancer [22]. Supplemental Table S2. Clinical details and IHC H-scores for breast tissuesa. [file 12943_2023_1861_MOESM2_ESM.docx]

**Supplemental Tables**

**Supplemental Table S1: Immunohistochemistry analysis of EYA4 expression in breast cancer (22).**

|  | Normal breast epithelium  n (%) | Breast carcinoma  n (%) | *P* value ^a^ |
| --- | --- | --- | --- |
| Negative | 3 (100) | 3 (25) | 0.0440 |
| Weak/moderate | 0 (0) | 9 (75) |  |
| Total | 3 (100) | 12 (100) |  |
| ^a^ Fisher's exact test | | | |

**Supplemental Table S2: Clinical details and IHC H-scores for breast tissues ^a^.**

| Patient ID | Tissue type | Sex | H-score |
| --- | --- | --- | --- |
| 2773 | Normal breast | Female | 20 |
| 3286 | Normal breast | Female | 85 |
| 3544 | Normal breast | Female | 80 |
| 1775 | Ductal breast carcinoma | Female | 100 |
| 1785 | Ductal breast carcinoma | Female | 110 |
| 1874 | Ductal breast carcinoma | Female | 80 |
| 1910 | Ductal breast carcinoma | Female | 155 |
| 1916 | Ductal breast carcinoma | Female | 180 |
| 1939 | Ductal breast carcinoma | Female | 170 |
| 2091 | Ductal breast carcinoma | Female | 90 |
| 2160 | Ductal breast carcinoma | Female | 110 |
| 2428 | Ductal breast carcinoma | Female | 180 |
| 2565 | Lobular breast carcinoma | Female | 100 |
| 2805 | Lobular breast carcinoma | Female | 100 |
| 3546 | Lobular breast carcinoma | Female | 80 |
| **^a^** All clinical data are from the Human Protein Atlas (22) (https://www.proteinatlas.org) | | | |
